# Breaking Barriers to High‐Practical Li‐S Batteries with Isotropic Binary Sulfiphilic Electrocatalyst: Creating a Virtuous Cycle for Favorable Polysulfides Redox Environments

**DOI:** 10.1002/advs.202303916

**Published:** 2023-10-22

**Authors:** Wei Xiao, Kisoo Yoo, Jong‐Hoon Kim, Hengyue Xu

**Affiliations:** ^1^ Department of Mechanical Engineering Yeungnam University 280 Daehak‐ro Gyeongsan‐si Gyeongsanbuk‐do 38541 South Korea; ^2^ Energy Storage and Conversion Laboratory Department of Electrical Engineering Chungnam National University Daejeon 34134 Republic of Korea; ^3^ Institute of Biopharmaceutical and Health Engineering Tsinghua Shenzhen International Graduate School Tsinghua University Shenzhen 518055 China

**Keywords:** binary sulfiphilic catalyst, isotropic adsorption‐catalysis, practical Li–S batteries, virtuous cycle, wide‐temperature range application

## Abstract

Investigations into lithium–sulfur batteries (LSBs) has focused primarily on the initial conversion of lithium polysulfides (LiPSs) to Li_2_S_2_. However, the subsequent solid–solid reaction from Li_2_S_2_ to Li_2_S and the Li_2_S decomposition process should be equally prioritized. Creating a virtuous cycle by balancing all three chemical reaction processes is crucial for realizing practical LSBs. Herein, amorphous Ni_3_B in synergy with carbon nanotubes (aNi_3_B@CNTs) is proposed to implement the consecutive catalysis of S_8(solid)_ → LiPSs_(liquid)_ → Li_2_S_(solid)_ →LiPSs_(liquid)_. Systematic theoretical simulations and experimental analyses reveal that aNi_3_B@CNTs with an isotropic structure and abundant active sites can ensure rapid LiPSs adsorption‐catalysis as well as uniform Li_2_S precipitation. The uniform Li_2_S deposition in synergy with catalysis of aNi_3_B enables instant/complete oxidation of Li_2_S to LiPSs. The produced LiPSs are again rapidly and uniformly adsorbed for the next sulfur evolution process, thus creating a virtuous cycle for sulfur species conversion. Accordingly, the aNi_3_B@CNTs‐based cell presents remarkable rate capability, long‐term cycle life, and superior cyclic stability, even under high sulfur loading and extreme temperature environments. This study proposes the significance of creating a virtuous cycle for sulfur species conversion to realize practical LSBs.

## Graphical abstract

Virtuous cycle for consecutive electrocatalysis. Specifically, the binary sulfiphilic aNi_3_B with isotropic structure enables a rapid/uniform LiPSs adsorption. Meanwhile, the superior catalytic capability and ionic/electronic conductivity synergistically facilitated the fast/homogeneous Li_2_S precipitation. Uniform Li_2_S deposition helps fully oxidize it to LiPSs, which are quickly and uniformly adsorbed for the next sulfur evolution, creating a virtuous cycle for sulfur species conversion.

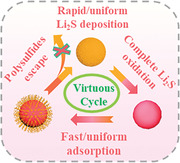

## 1 Introduction

Lithium sulfur batteries (LSBs) have captured tremendous attention for next‐generation energy‐storage devices owing to the low cost, naturally abundant of raw material and high energy density of 2600 Wh kg^−1^, which is several times higher than that of state‐of‐the‐art lithium‐ion batteries.^[^ ^1^, ^2^ ^]^ However, their large‐scale commercialization is hindered by the sluggish redox kinetics and rampant shuttling behavior of lithium polysulfides (LiPSs), which severely restrict sulfur utilization and deteriorate capacity cyclic stability.^[^ ^3^, ^4^ ^]^ During the sulfur reduction reaction (SRR), the escape of LiPSs into the electrolyte and their slow reduction kinetics result in the gradual degradation of both electrodes as well as electrolyte contamination. Meanwhile, insulating Li_2_S tends to aggregate over the electrocatalyst surface, thus creating an unfavorable environment for the subsequent sulfur evolution reaction (SER).^[^ ^5^, ^6^ ^]^ In the upcoming SER, severe Li_2_S aggregation and high Li_2_S activation energy barriers directly result in insufficient Li_2_S oxidation.^[^ ^7^, ^8^ ^]^ The repetition of these unfavorable behaviors creates a vicious cycle for sulfur species conversion, which severely exacerbates their reaction environment, thus resulting in significant losses of the active species and rapid capacity decay during the cyclic process.

Over the past decade, the MX host material (where M represents the metallic element and X denotes the non‐metallic element) played a leading role in suppressing the shuttle behavior of polysulfides, including metal oxides,^[^ ^9^ ^]^ sulfides,^[^ ^10^ ^]^ nitrides,^[^ ^11^ ^]^ and phosphides.^[^ ^12^ ^]^ MX materials with both lithiophilic and sulfiphilic sites can chemically anchor LiPSs via M‐S or X‐Li bonding. However, the presence of robust Li‐X bond can impede the direct electron transfer to polysulfides, which in turn may decelerate the Li^+^ diffusion rate. The limited supply of Li^+^ decelerates the reaction kinetics of sulfur species during both SRR and SER processes, which may not truly prevent the system from undergoing the vicious cycle mentioned above.^[^ ^13^ ^]^ Unlike MX‐based materials, which only exhibit single metal−sulfur absorption, the boron sites in metal borides (MBs) with empty orbits along with the metal−sulfur absorption effect can absorb the polysulfides effectively, thus enabling a dual‐site adsorption to anchor polysulfides through both M‐polysulfides and B‐polysulfides interactions.^[^ ^14^ ^]^ These binary sulfiphilic interactions simultaneously enable sufficient LiPSs anchoring and rapid Li^+^ transport. Meanwhile, the high electrocatalytic capability of MB can simultaneously accelerate the reaction kinetics of sulfur species during both SRR and SER processes.^[^ ^15^ ^]^

Based on recent publications, most catalytic materials for LSBs are based on their crystalline structures.^[^ ^14^, ^16^ ^]^ However, three limitations of crystalline materials must be considered. First, their structural anisotropy is not conducive to the maximum exposure of active sites with optimized orientations. The anisotropic nature of the exposed crystalline facets results in host material exhibiting non‐uniform adsorption‐catalysis toward LiPSs. This leads to the escape of LiPSs and significant residual of Li_2_S in the weakly catalytic facets, which may cause the system to fall into the vicious cycle. Second, the intrinsically limited exposed active sites in bulky particles restrict the adsorption‐catalysis capability toward sulfur species.^[^ ^17^ ^]^ Additionally, fabricating MBs with task‐specific morphologies is extremely challenging due to the complex synthesis conditions of crystalline MBs. Compared with crystalline materials, amorphous materials present superior advantages, including the isotropic structure, abundant defects and active sites. More importantly, the amorphous electrocatalyst possesses remarkable flexibility in geometry when interacting with the reacting molecules, which enables them to self‐regulate themselves according to the electrocatalytic condition^[^ ^18^ ^]^ Exploiting this property, the amorphous MB consistently exposes the highest catalytic facet with optimized orientation for LiPSs electrocatalysis, resulting in uniform and efficient adsorption‐catalysis toward the LiPSs redox process. Consequently, the uniform adsorption‐catalysis further lead to uniform Li_2_S precipitation and instant activation of Li_2_S. The produced LiPSs are again rapidly and uniformly adsorbed for the next sulfur evolution process, thus creating a virtuous cycle for the future sulfur species conversion. Based on theoretical analyses, MBs with an amorphous structure may be a promising candidate for preventing the system from entering such a vicious cycle. However, to the best of our knowledge, the application of amorphous MB material on LSBs as host material has been rarely investigated.

Based on this analysis, we herein utilize amorphous Ni_3_B nanoparticles in synergy with carbon nanotubes (aNi_3_B@CNTs) as a regulator to create a virtuous cycle for sulfur species conversion. Both experiments and theoretical calculations reveal the superiority of amorphous Ni_3_B in the adsorption–diffusion catalysis of LiPSs. In detail: 1) The binary sulfiphilic Ni and B sites in Ni_3_B enable the dual‐site adsorption of LiPSs through B‐polysulfide and Ni‐polysulfide. The high anchoring site density in synergy with isotropic property ensures a rapid/uniform adsorption toward LiPSs during both SRR and SER. 2) The amorphous Ni_3_B possesses high catalytic activity and abundant catalytically active sites, which maximizes the adsorption–catalytic effect for the rapid reduction of LiPSs during the SRR. 3) The aNi_3_B@CNTs demonstrates superior catalysis for Li_2_S decomposition during the SER by weakening the Li─S bond energy. 4) The CNTs conductive network enables a homogeneous distribution of Ni_3_B particles and simultaneously provides long‐range ordered electron conductivity for fast electron transfer during the LiPSs redox process. 5). The aNi_3_B@CNTs composite can be synthesized via a facile aqueous reduction process without heating process, which makes it a promising option for the large‐scale commercial application of lithium‐sulfur batteries. Such a synergistic effect during both the SRR and SER creates a virtuous cycle for sulfur species conversion, thereby enabling a favorable environment for their reaction during the cycling process. Benefiting from this virtuous cycle, the aNi_3_B@CNTs‐based cell demonstrates state‐of‐the‐art electrochemical performance. Specifically, the aNi_3_B@CNTs‐based cell shows superior rate capability (479.14 mAh g^−1^ at 8 C) and an ultralong cycling life at high C‐rate of 5 C (low capacity decay of 0.028% per cycle over 2000 cycles). Under extreme temperature of 0 and 50 °C, the aNi_3_B@CNTs‐based cell exhibits outstanding capacity degradation of 0.059% and 0.076% at 2 C for 300 cycles, which are similar to the degradation rate observed at room temperature (0.027%). Even under a high sulfur loading of 10.45 mg cm^−2^, the aNi_3_B@CNTs‐based cell retains an ultrahigh areal capacity of 7.52 mAh cm^−2^, providing insights for future practical application.

## 2 Result and Discussion

### 2.1 Microstructure and Component Analysis of aNi_3_B@CNTs Electrocatalysts

**Figure 1:**
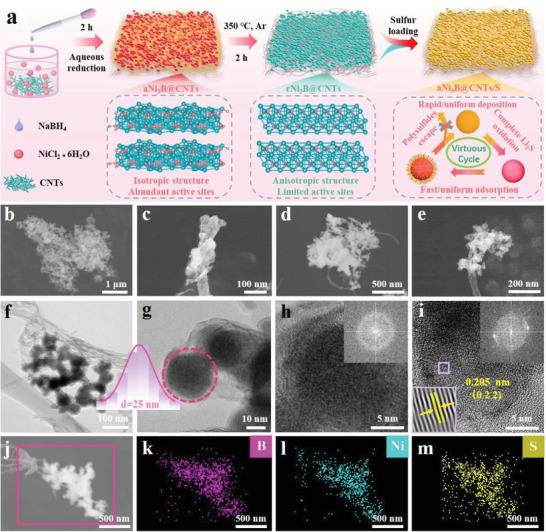
a) Schematic illustration of a/cNi_3_B@CNTs/S fabrication process. SEM images of b,c) aNi_3_B@CNTs, and d,e) aNi_3_B@CNTs/S. TEM images of f,g) aNi_3_B@CNTs. Inset: the corresponding size distribution of aNi_3_B nanoparticles. HRTEM images of h) aNi_3_B@CNTs, and i) cNi_3_B@CNTs. Inset: The corresponding fast Fourier transform (FFT) pattern. j) SEM image of aNi_3_B@CNTs/S and the related elemental mapping of k) boron, l) nickel, and m) sulfur.

In this study, an isotropic aNi_3_B@CNTs featuring abundant active sites and conductive surfaces was rationally designed and synthesized via a facile aqueous reduction process (**Figure** **1**a). Benefitting from the unique binary sulfiphilic adsorption and isotropic properties, when serving as a host material, the aNi_3_B@CNTs affords a rapid/homogeneous adsorption–catalysis process toward sulfur species, thereby creating a virtuous cycle during the entire charge/discharge process. The microstructures of various materials have been investigated via scanning electron microscopy (SEM) and transmission electron microscopy (TEM). As shown in Figure 1b,c, amorphous Ni_3_B appeared as cloud‐like nanoparticles that were dispersed and tightly coupled throughout the CNTs network. The TEM image further reveal its cloudlike nanoparticle characteristic and their particle diameter of ≈25 nm, as shown in Figure 1f,g. Additionally, the amorphous nature of aNi_3_B is evidenced by the lack of observable crystalline fringes (Figure 1h). Although crystalline Ni_3_B features a cloud structure similar to that of amorphous Ni_3_B (Figure 1i), the presence of distinct lattice fringes suggests the crystalline nature of cNi_3_B. More specifically, the lattice spacing of 0.205 nm corresponds to the (0 2 2) plane of cNi_3_B, and the clear diffraction rings related to crystalline Ni_3_B are observed in the related fast Fourier transform (FFT) pattern. After sulfur impregnation, the surface of aNi_3_B@CNTs/S became smooth, indicating the successful loading of sulfur (Figure 1d,e). Moreover, the elemental mapping images revealed a uniform elemental distribution within aNi_3_B@CNTs/S, thus further demonstrating the homogeneous loading of sulfur (Figure 1k–m).

**Figure 2:**
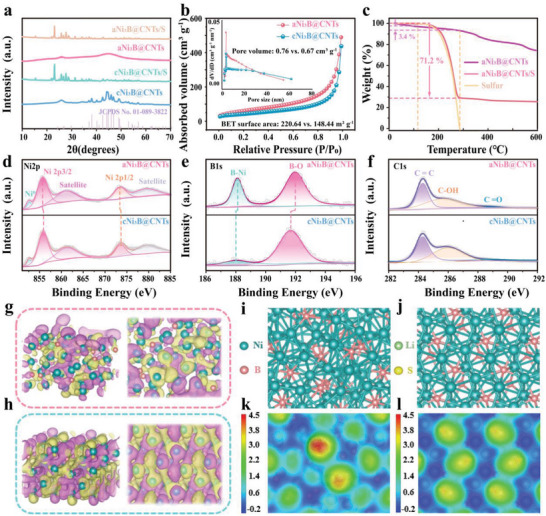
a) XRD patterns of aNi_3_B@CNTs, cNi_3_B@CNTs, aNi_3_B@CNTs/S, and cNi_3_B@CNTs/S. b) N_2_ adsorption/desorption isotherms and pore size distribution (inset) of aNi_3_B@CNTs and cNi_3_B@CNTs. c) TGA curves of aNi_3_B@CNTs and aNi_3_B@CNTs/S. XPS spectra of d) Ni2p, e) B1s, and f) C1s from aNi_3_B@CNTs and cNi_3_B@CNTs. Electronic orbital distribution in g) aNi_3_B, and h) cNi_3_B. Optimized structure of i) aNi_3_B and j) cNi_3_B. Calculated electron‐density isosurface for k) aNi_3_B, and l) cNi_3_B. Electron‐density isosurface was plotted at 0.01 e Bohr^−3^. Color bar represents electrostatic potential scale.

X‐ray diffraction (XRD) was employed to identify the phases of prepared samples. As displayed in **Figure** **2**a, both samples exhibit an intense diffraction peak at 2θ = 26.3°, which corresponds to the graphitic (0 0 2) plane of the conductive CNTs skeletons. In addition, a broad peak at ≈45° can be observed in the diffraction pattern of aNi_3_B@CNTs, which indicates the amorphous nature of Ni_3_B in the composites. By contrast, the cNi_3_B@CNTs exhibited distinct diffraction peaks of Ni_3_B (JCPDS no. 01‐089‐3822), confirming the highly crystalline property of Ni_3_B in the cNi_3_B@CNTs composites. After sulfur impregnation, both aNi_3_B@CNTs/S and cNi_3_B@CNTs/S exhibited the typical sulfur diffraction peaks, verifying the successful impregnation of sulfur. Additionally, the aNi_3_B@CNTs sample exhibited a higher Brunauer–Emmett–Teller (BET) specific surface area and pore volume of 220 m^2^ g^−1^ and 0.76 cm^3^ g^−1^ than cNi_3_B@CNTs (148.44 m^2^ g^−1^ and 0.67 cm^3^ g^−1^, respectively, as shown in Figure 2b). The higher specific area and pore volume were endowed by the amorphous nature of aNi_3_B, which provides more catalytically active sites for maximizing the adsorption–catalytic effect of aNi_3_B@CNTs toward LiPSs redox reaction. The specific surface area and pore volume of both samples significantly decreased after loading sulfur (Figure S2a,b, Supporting Information), further confirming successful sulfur integration. Thermogravimetric analysis (TGA) reveals a high actual sulfur content of 70.2% in the aNi_3_B@CNTs/S, which is calculated based on a previously reported method,^[^ ^19^ ^]^ as illustrated in Figure 2c.

X‐ray photoelectron spectroscopy (XPS) was employed to investigate the surface chemistry. Figure 2d shows the Ni2p spectrum of the aNi_3_B@CNTs and cNi_3_B@CNTs. In the case of the aNi_3_B@CNTs, the peak at 852.2 eV is attributed to metallic Ni^0^ arriving from the inner Ni_3_B nanoparticles.^[^ ^20^ ^]^ In addition to satellite peaks, the aNi_3_B@CNTs presented two peaks centered at 856.28 and 874.18 eV for Ni 2p_3/2_ and Ni 2p_1/2_, respectively, which are assigned to the Ni─B bonds of aNi_3_B.^[^ ^21^ ^]^ Two distinct peaks could be observed in the B1s spectra of both samples (Figure 2e). The peak at 187.7 eV can be attributed to the B─Ni bonds in aNi_3_B, whereas the peak at a higher binding energy 191.7 eV can be associated with B─O bond in borate or boron oxide due to the surface oxidation of Ni_3_B after being exposed to air.^[^ ^22^ ^]^ Compared with crystalline Ni_3_B, the Ni‐B peak in Ni2p spectrum shifted to a lower binding energy, whereas the B‐Ni peak in B1s spectrum shifted to a higher binding energy, indicating the presence of numerous unsaturated bonds in the amorphous Ni_3_B.^[^ ^23^ ^]^ Figure 2f shows the C1s spectra of the aNi_3_B@CNTs and cNi_3_B@CNTs; both spectra can be deconvoluted into three peaks. The peaks located at 284.26, 285.98, and 288.79 eV were ascribed to C = C, C‐OH, and C = O, respectively.^[^ ^24^ ^]^

Density‐functional theory (DFT) calculations were conducted to fain further insight into the superiority of amorphous Ni_3_B. The spatial structure of the bonding and antibonding orbitals near the Fermi level were investigated for both aNi_3_B (Figure 2g) and cNi_3_B (Figure 2h). The crystalline Ni_3_B exhibited regular spatial structure distributions for the molecular orbitals near the Fermi level. However, in the case of amorphous Ni_3_B, the spatial structure distributions of the orbitals near the Fermi level exhibited an obvious disordered, suggesting that the surface charge density distributions were reconstructed significantly to activate the electrocatalytic activity of aNi_3_B. Moreover, the electronic interactions and chemisorption behaviors of the two surface configurations were evaluated based on electrostatic potential surfaces. As shown in Figure 2i,k, compared with cNi_3_B, aNi_3_B exhibited a distinct red region, which indicates a higher electrostatic potential and lower electron density. This is attributed to electron redistribution caused by the amorphous structure. The absence of electrons induced disordered Ni_3_B, which pulled electrons from the polysulfide anions, thus resulting in a stronger adsorption ability and lower energy barriers for polysulfide conversion.^[^ ^25^, ^26^ ^]^ These results demonstrate the superior adsorption‐catalysis capability of amorphous Ni_3_B for polysulfide conversion.

### 2.2 Absorption and Catalytic Mechanism of LiPSs

**Figure 3:**
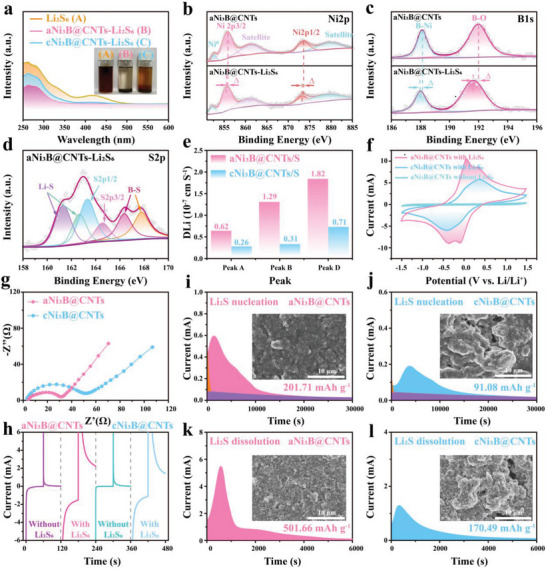
a) UV–vis spectra and photograph (inset) of Li_2_S_6_ solution before and after adding aNi_3_B@CNTs and cNi_3_B@CNTs. XPS spectra of b) Ni2p, and c) B1s from aNi_3_B@CNTs and aNi_3_B@CNTs–Li_2_S_6_. d) S2p spectra of aNi_3_B@CNTs–Li_2_S_6_. e) Calculated Li‐ion diffusion coefficient (D_Li_^+^) comparison. f) CV profiles of Li_2_S_6_‐symmetrical cells at 10 mV s^−1^, and g) Nyquist plots of Li_2_S_6_‐symmetric cells. h) Chronoamperometric curves of Li_2_S_6_‐symmetric cells. Potentiostatic discharge profiles of Li_2_S nucleation for cells with i) aNi_3_B@CNTs and j) cNi_3_B@CNTs. Insets show SEM images of electrode after Li_2_S nucleation. Potentiostatic charge profiles of Li_2_S dissolution with k) aNi_3_B@CNTs and l) cNi_3_B@CNTs. Insets show SEM images of electrode after Li_2_S dissolution.

The superior adsorption–catalytic capability of the disordered Ni_3_B was further verified through experiments. To investigate the adsorption capability, the adsorption experiments were conducted via soaking the aNi_3_B@CNTs and cNi_3_B@CNTs individually into Li_2_S_6_ solution. The solutions with aNi_3_B@CNTs added became almost colorless after 12 h (**Figure** **3**a), whereas the color of the solution containing cNi_3_B@CNTs became slightly lighter, indicating stronger adsorption capability of amorphous Ni_3_B. It should be noted that the crystalline Ni_3_B also possesses binary sulfiphilic sites for anchoring LiPSs, which can enable the satisfactory adsorption activity toward LiPSs. However, the majority of active sites for adsorption remain embedded inside the bulky particles owing to the crystalline structure, resulting in the reduced adsorption‐catalysis capability. The amorphous structure perfectly compensates for this defect and simultaneously endows it with a strong adsorption capacity and rich anchoring sites. Furthermore, the supernatants were investigated via UV–vis spectroscopy. The absorbance peak corresponding to Li_2_S_6_ nearly vanished for the aNi_3_B@CNTs‐Li_2_S_6_ solution, whereas that of the cNi_3_B@CNTs–Li_2_S_6_ solution only weakened, indicating the superior adsorption capability of aNi_3_B@CNTs toward LiPSs.

XPS analysis was performed to further clarify the interactions (Figure 3b,c). After adsorption, the Ni2p spectrum of aNi_3_B@CNTs–Li_2_S_6_ powder shifted to a lower binding energy, indicating the transfer of electrons from the polysulfide anions to the positively charged Ni.^[^ ^27^ ^]^ This phenomenon was also observed in the B 1s spectrum, which can be attributed to the electron‐deficient structure of B atoms, allowing them to accept additional electrons from the polysulfides anions.^[^ ^28^ ^]^ A similar downward shift in binding energy was observed in the Ni2p and B1s spectra of the cNi_3_B@CNTs–Li_2_S_6_ sample. Therefore, the downshift quantities (Δ) of the two samples were recorded and compared. As shown in Figure S5 (Supporting Information), significantly higher downshifts were observed for the aNi_3_B@CNTs–Li_2_S_6_ sample, indicating a significant increase in the amount of electron transfer from the S atoms of Li_2_S_6_ to the Ni and B atoms. This provides additional evidence that verifies the stronger interaction of amorphous Ni_3_B with polysulfide anions, which is consistent with the DFT simulation prediction discussed above (Figure 2k,l). Moreover, the B─S bond was detected in the B1s spectra of aNi_3_B@CNTs–Li_2_S_6_ (Figure 3d), further confirming the sulfiphilic anchoring sites of B in the aNi_3_B@CNTs host material.

Apart from the unique dual‐site adsorption capability toward LiPSs, the aNi_3_B@CNTs was superior in promoting the diffusion of Li^+^. To verify this, CV measurements were conducted at different scan rates to probe the LiPSs diffusion behavior, as shown in Figure S6a–f (Supporting Information). The Li^+^ diffusion coefficient (D_Li_^+^) was qualitatively determined based on the Randles–Sevcik equation. As shown in Figure 3e, the aNi_3_B@CNTs‐based cell exhibited demonstrated a significantly greater quantity of D_Li_^+^, suggesting a faster Li^+^ diffusion efficiency. More importantly, the aNi_3_B@CNTs composites exhibited excellent electrocatalytic capabilities for accelerating polysulfide conversion. The electrocatalytic capabilities was identified by CV measurement of symmetric cells. As shown in Figure 3f, the aNi_3_B@CNTs‐based cell demonstrated a clear anodic peak separation, higher peak current, and smaller potential gap compared with the cNi_3_B@CNTs‐based cell, suggesting the superior catalytic capability of amorphous Ni_3_B toward the reaction of sulfur species. Additionally, the CV profile of aNi_3_B@CNTs retained consistent in amplitude after four cycles (Figure S7, Supporting Information), suggesting the high catalytic stability of amorphous Ni_3_B. The Nyquist plot in Figure 3g reveals a smaller semicircle diameter, confirming that the redox kinetics of LiPSs were significantly promoted due to the presence of amorphous Ni_3_B.^[^ ^23^, ^29^ ^]^ Figure 3h presents the chronoamperometry curves of the symmetrical cells. Without the addition of Li_2_S_6_ into electrolyte, no current response was observed in both aNi_3_B@CNTs and cNi_3_B@CNTs symmetric cell, and both of the chronoamperometry curves exhibited an apparent current response after the addition of Li_2_S_6_, indicating that the majority of response current was generated by the lithiation/delithiation process instead of double layer capacitance.^[^ ^30^ ^]^ Moreover, the aNi_3_B@CNTs‐based cell exhibited higher current responses than cNi_3_B@CNTs. This indicates faster redox kinetics of Li_2_S_6_ were achieved in aNi_3_B@CNTs symmetric cell, suggesting the considerable superior electrocatalytic activity of amorphous Ni_3_B than crystalline Ni_3_B.

In addition to the adsorption‐diffusion‐catalysis of LiPSs, the deposition of produced Li_2_S and subsequent decomposition reactions are another crucial component in the reaction cycle of sulfur species. Potentiostatic Li_2_S precipitation and dissolution experiments were conducted to investigate the Li_2_S deposition and activation behavior in the presence of aNi_3_B or cNi_3_B. As presented in Figure 3i,j, the aNi_3_B@CNTs‐based cell demonstrated a much stronger current response and a higher Li_2_S precipitation capacity than its crystalline counterparts (201.71 *vs*. 91.08 mAh g^−1^), suggesting that the aNi_3_B electrocatalyst significantly promote the Li_2_S precipitation kinetics.^[^ ^31^, ^32^ ^]^ Furthermore, the ex situ SEM was utilized to observe the deposition morphology of Li_2_S. As depicted in the insets of Figure 3i,j, a uniform Li_2_S precipitation was observed in the aNi_3_B@CNTs electrode surface, which in in stark contrast to the significant Li_2_S aggregation observed in cNi_3_B@CNTs. The rapid/uniform deposition of Li_2_S created a favorable condition for the subsequent rapid dissociation‐oxidation of produced Li_2_S. To verify this analysis, the decomposition behavior of the deposited Li_2_S was evaluated via a potentiostatic charge test. As expected, the aNi_3_B@CNTs‐based cell exhibited a substantially higher Li_2_S decomposition capacity (501.66 mAh g^−1^) than the Ni_3_B@CNTs‐based cell (170.49 mAh g^−1^), as shown in Figure 3k,l. Furthermore, SEM images suggested that the Li_2_S precipitation persisted on the cNi_3_B@CNTs electrode surface after charging process, in contrast to the clean surface of aNi_3_B@CNTs electrode (inset of Figure 3k,l). Benefitting from the high geometry flexibility with the reacting molecules, the amorphous Ni_3_B host material can enable them to self‐regulate themselves according to the electrocatalytic condition, and consistently exposes the highest catalytic facet for LiPSs adsorption‐electrocatalysis, which enables a rapid/uniform Li_2_S deposition and an instant/complete Li_2_S dissolution, thereby creating a virtuous cycle for sulfur species conversion.

### 2.3 DFT Theoretical Simulation Analysis

**Figure 4:**
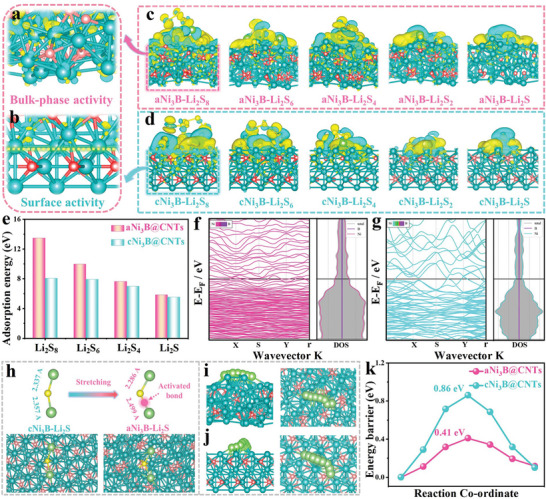
Charge density difference patterns of a) aNi_3_B and b) cNi_3_B after adsorption. Yellow distribution represents charge accumulation, while the blue distribution represents charge depletion. The difference in charge after binding with sulfur species on surfaces of c) aNi_3_B and d) cNi_3_B. e) Adsorption energy comparison; f) Band structure and density of states (DOS) of aNi_3_B and cNi_3_B; h) schematic illustration of Li_2_S adsorbed on aNi_3_B and cNi_3_B surface and length of Li‐S bond; Schematic diagrams of Li^+^ migration pathways on i) aNi_3_B and j) cNi_3_B surfaces; k) the energy barrier for Li^+^ diffusion on aNi_3_B and cNi_3_B surfaces.

A series of theoretical calculations was performed to investigate the adsorption‐diffusion‐catalysis capability from the molecular level. The charge density difference offers intuitive insights into the bonding interactions between the host and sulfur species. As shown in **Figure** **4**a, while absorbing sulfur species, charge accumulation and depletion could be observed among the entire amorphous Ni_3_B structure; this indicates the bulk‐phase catalytic activity of aNi_3_B, which can be ascribed to electronic redistribution in the amorphous structure. By contrast, charge accumulation and depletion were only observed near the adsorption surface in the case of crystalline Ni_3_B (Figure 4b), verifying the limited surface catalytic activity due to the fact that the majority of active sites were embedded inside the bulky crystalline particles, which considerably restricted their anchor‐catalysis capability toward sulfur species. Moreover, the amorphous Ni_3_B exhibited a more intense charge exchange than its crystalline counterpart while anchoring sulfur species, as shown in Figure 4c,d, verifying the higher anchor‐catalysis activity of amorphous Ni_3_B. The adsorption energies between Li_2_S_8_, Li_2_S_6_, Li_2_S_4_, and Li_2_S molecules and the surfaces of the amorphous Ni_3_B surface were −13.47, −9.98, −7.63, and −5.86 eV, respectively, which were higher than those (−8.07, −7.93, −6.99, and −5.53 eV, respectively) on the crystalline Ni_3_B surface, as shown in Figure 4e. Interestingly, although crystalline Ni_3_B exhibited a theoretically satisfactory adsorption energy, it showed the worst experimental adsorption behavior for Li_2_S_6_ (as discussed in Figure 3a). This disparity can be attributed to the bulk of crystalline Ni_3_B exposing a limited number of active sites in comparison to amorphous Ni_3_B, which agrees with our analysis above (Figure 4a,b).

The band structure and density of states (DOS) plot reveals that both aNi_3_B and cNi_3_B exhibited electrical conductivity without any energy gap near the Fermi level. As shown in Figure 4f,g, the DOS of the aNi_3_B@CNTs showed greater delocalization near the Fermi level than its crystalline counterpart, suggesting a higher electrical conductivity for amorphous Ni_3_B. Moreover, amorphous Ni_3_B is conducive to lower the Li_2_S nucleation and decomposition barrier, thereby enabling rapid Li_2_S precipitation and instant Li_2_S decomposition. As shown in Figure 4h, one of the Li─S bonds on the amorphous Ni_3_B surface is significantly longer than that on the crystalline Ni_3_B surface, which can serve as an activated bond for rapid Li_2_S diffusion and decomposition. Benefiting from the accelerated redox process of Li_2_S, both the shuttle behavior of the LiPSs during the discharge and the Li_2_S residual during the charge process were simultaneously avoided; therefore, a virtuous cycle for sulfur species conversion could be created during the entire charge/discharge process. Figure 4i,j show the schematic illustrations of the Li^+^ diffusion pathways on the host surfaces. As shown in Figure 4k, the energy barrier for Li^+^ diffusion on the amorphous Ni_3_B was 0.41 eV, which was significantly lower than that on the crystalline Ni_3_B surface (0.86 eV). The lower energy barrier for diffusion suggests a higher Li^+^ diffusion rate, which is consistent with the experimental findings (Figure 3e).

### 2.4 Electrochemical Performance

**Figure 5:**
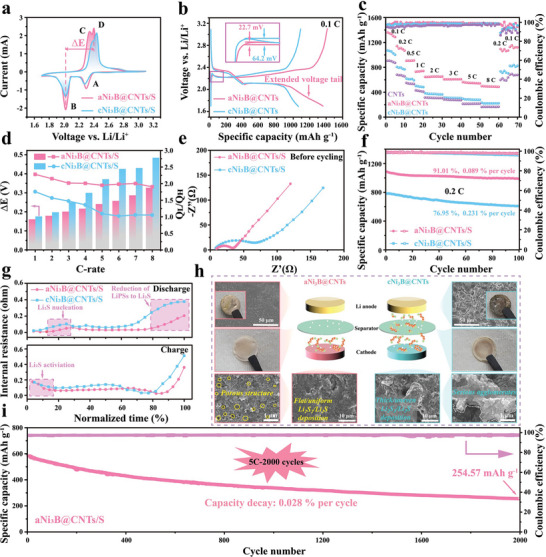
Electrochemical performance of aNi_3_B@CNTs and cNi_3_B@CNTs: a) CV profiles comparison at 0.1 mV s^−1^. b) Charge–discharge curves at the first cycle of 0.1 C. c) Rate performance comparison. d) Capacity ratio (Q_L_/Q_H_) and polarization potentials (ΔE) comparison at different C‐rates. e) EIS before cycling. f) Cyclic performance under 0.2 C within 100 cycles. g) Internal resistances relative to normalized discharge–charge time. h) Systematic post‐analysis of three crucial components of both cells. i) Comparison of long‐term cyclic stability at 5 C.

To verify the superiority of the aNi_3_B@CNTs in the conversion of sulfur species, a series of electrochemical analyses was conducted. The CV profiles of LSBs with both host materials at 0.1 mV s^−1^ are shown in **Figure** **5**a. Cathodic peaks A (2.30 V) and B (2.02 V) in the aNi_3_B@CNTs‐based cell are related to the multi‐reduction process from S_8_ to LiPSs and further reduction to insoluble Li_2_S_2_/Li_2_S, respectively. Another two anodic peaks (peak C at 2.32 V and peak D at 2.40 V) were ascribed to the multistep oxidation of Li_2_S_2_/Li_2_S to LiPSs and sulfur, respectively.^[^ ^33^ ^]^ Comparatively, the aNi_3_B@CNTs‐based cell possessed a stronger current response, clearly separated anodic peaks, a smaller potential gap (ΔE), and a higher/lower onset potential for reduction/oxidation peaks (Figures S8–S10, Supporting Information). These results verify the significantly accelerated redox kinetics of LiPSs owing to their unique dual‐site adsorption ability as well as the superior electrocatalytic capability of the aNi_3_B@CNTs.^[^ ^34^, ^35^ ^]^

The bidirectional catalytic capability of the aNi_3_B@CNTs was further investigated based on the charging/discharging behavior at 0.1 C. As shown in Figure 5b, the aNi_3_B@CNTs‐based cell exhibited two typical plateaus at ≈2.3 and 2.1 V, which can be attributed to the two‐step reduction from sulfur to Li_2_S. Moreover, the last sloping tail at the end of the discharge profile is related to a solid‐to‐solid reduction from Li_2_S_2_ to Li_2_S, which is accompanied by an increase in polarization. Comparatively, the aNi_3_B@CNTs‐based cell exhibited a much longer voltage tail than the cNi_3_B@CNTs‐based cell, demonstrating the superiority of amorphous Ni_3_B in accelerating the solid–solid reduction of Li_2_S_2_ to Li_2_S.^[^ ^36^ ^]^ This superior catalytic capability was similarly observed in the solid–liquid oxidation process. The inset of Figure 5b shows short voltage jumps in the charging curves, which correspond to Li_2_S activation during the conversion of Li_2_S_2_ to LiPSs. Clearly, the voltage jumps in the aNi_3_B@CNTs‐based cell (22.7 mV) were considerably lower than those of their crystalline counterparts (64.2 mV). This provides additional evidence that the aNi_3_B@CNTs efficiently accelerated the activation of Li_2_S during the conversion of Li_2_S to LiPSs.^[^ ^37^ ^]^

Benefitting from such a virtuous cycle for sulfur species conversion, excellent electrochemical performance can be expected. As shown in Figure 5c, the aNi_3_B@CNTs‐based cell delivered high specific capacities of 1361.02, 1141.57, 905.03, 729.43, 644.23, 607.24, 562.37, and 497.14 mAh g^−1^ at 0.1, 0.2, 0.5, 1.0, 2.0, 3.0, 5.0, and 8.0 C, respectively, which were apparently superior to those of CNTs‐based and cNi_3_B@CNTs‐based cell. Additionally, the capacity was recovered to 1127.36 mAh g^−1^ after the current was returned from 8.0 to 0.1 C, achieving a recovery of 93.33% based on initial capacity, which implies superior rate capability and reversibility of the aNi_3_B@CNTs‐based cell. Without the addition of aNi_3_B or cNi_3_B catalyst, the pure CNTs host material lacks the efficient anchoring ability and catalytic capability toward the redox process of LiPSs, which consequently results in a significant loss of active material and deteriorated rate capability in CNTs‐based cell. To further explored the superiority of amorphous Ni_3_B, the galvanostatic charge‐discharge profiles of both aNi_3_B@CNTs and cNi_3_B@CNTs at different C‐rates were further compared and analyzed, as shown in Figure S11 (Supporting Information). The ratio of capacity at low discharge plateau to that at high plateau (Q_L_/Q_H_) is a key reference point for evaluating the LiPSs redox efficiency.^[^ ^38^ ^]^ As displayed in Figure 5d, the cNi_3_B@CNTs/S displayed a significantly lower Q_L_/Q_H_ ratio (< 2.0) at each C‐rate. And the value of Q_L_/Q_H_ decreased significantly to merely ≈1.0 when the discharge C‐rate reach up to 2 C, indicating an inferior conversion efficiency in the liquid‐solid reduction of LiPSs to Li_2_S_2_/Li_2_S, particularly at high C‐rates. The limited active sites of the cNi_3_B@CNTs for anchoring and catalysis should be responsible for the rampant shuttle behavior of LiPSs and their low conversion efficiency. By contrast, the aNi_3_B@CNTs‐based cell exhibited a considerably higher Q_L_/Q_H_ ratio at each C‐rate and maintained a high ratio of ≈2.0, even at each ultrahigh C‐rate. Synergetic with the smaller potential polarization (ΔE) observed in the aNi_3_B@CNTs‐based cell at each C‐rate, these analyses provide strong evidence to verify the highly efficient anchor‐catalysis toward LiPSs conversion with the help of amorphous Ni_3_B.^[^ ^39^, ^40^, ^41^ ^]^

The accelerated conversion kinetics of LiPSs on the aNi_3_B@CNTs electrode was verified via electrochemical impedance spectroscopy (EIS) (Figure 5e; Figures S12 and S13, Supporting Information). Table S1 (Supporting Information) summarizes the electrochemical parameters *R*_ct_ and *R*_e_ obtained from fitting. Both before and after cycling, the *R*_ct_ values of the aNi_3_B@CNTs electrode are significantly lower, demonstrating rapid charge transfer between the electrode and electrolyte. Additionally, Rs refers to the Li_2_S/Li_2_S_2_ insulation layer impedance. After cycling, the aNi_3_B@CNTs‐based cell displayed a lower Rs, indicating an acceleration in the kinetics of the transition from Li_2_S/Li_2_S_2_ to S_8_. Besides with the superior rate performance, the aNi_3_B@CNTs‐based cell exhibited impressive cyclic stability as well. As presented in Figure 5f, the aNi_3_B@CNTs‐based cell exhibited a higher initial specific capacity of 1085.08 mAh g^−1^ at 0.2 C, and retains a remarkable capacity of 987.54 mAh g^−1^ after 100 cycles, which suggests a considerably higher capacity retention of 91.01% than cNi_3_B@CNTs‐based cell (76.95%), suggesting the superior cyclic stability of aNi_3_B@CNTs‐based cell. At the reaction interface of the cNi_3_B@CNTs, the structural anisotropy of the bulky particles significantly hindered the maximum exposure of the active sites. Insufficient active sites resulted in the escape of LiPSs that could not be anchored by the host material, which consequently resulted in the rampant shuttling behavior of the LiPSs. The real‐time internal resistances of batteries were further analyzed through the galvanostatic intermittent titration technique (GITT). As shown in Figure 5g and Figure S14 (Supporting Information), the aNi_3_B@CNTs‐based cell exhibited an obviously smaller internal resistance (R_Ω_) than its cNi_3_B@CNTs counterpart at the reduction from LiPSs into Li_2_S_2_/Li_2_S as well as the nucleation/activation points of Li_2_S (shown in the dotted box), confirming the bidirectional catalytic capability of amorphous Ni_3_B on these three crucial processes.^[^ ^42^ ^]^

To further probe the mechanism of capacity fading, the coin cell was disassembled, and three key components were analyzed. As shown in Figure 5h, after being fully discharged for 100 cycles, the cNi_3_B@CNTs/S cathode showed a thick and uneven Li_2_S_2_/Li_2_S film with severe agglomeration. This can be attributed to the structural anisotropy of the crystalline Ni_3_B. In detail, the lattice planes exposed on each surface of the crystalline Ni_3_B particles were limited and different, and thus demonstrated limited and considerably different adsorption and catalytic capabilities toward LiPSs; therefore, a thick and inhomogeneous layer of Li_2_S_2_/Li_2_S was deposited in the cNi_3_B@CNTs cathode. Furthermore, the thick Li_2_S_2_/Li_2_S film covered the active sites and hindered the efficient contact between the conductive substrate and electrolyte, which significantly worsened the reaction environment for future cyclic processes. Moreover, the size of the Li_2_S_2_/Li_2_S agglomerates exceeded the threshold of the electron tunneling thickness.^[^ ^43^ ^]^ These solid products were difficult to be reused owing to the poor electrical contact, thus resulting in the inadequate Li_2_S_2_/Li_2_S conversion and loss of the active material. Such a vicious cycle ultimately degraded the reaction environment and resulted in severe capacity fading during the cyclic process. By contrast, a flat and uniform deposition of Li_2_S_2_/Li_2_S was observed on the cathode surface of the aNi_3_B@CNTs‐based cell, which featured numerous holes. The isotropic structure with abundant active sites enabled the homogeneous adsorption of LiPSs and rapid Li_2_S_2_/Li_2_S deposition.^[^ ^44^ ^]^ The uniform Li_2_S_2_/Li_2_S deposition and numerous holes on the surface provided good electric contact for rapid Li_2_S decomposition, thereby facilitating the following Li_2_S decomposition and LiPSs adsorption–catalysis process, which created a virtuous cycle to well maintain a favorable environment for the future adsorption and catalysis of LiPSs.^[^ ^45^ ^]^

Such a favorable reaction environment was verified by the conditions observed in the anode and separator. As displayed in Figure 5h and Figure S15 (Supporting Information), the cycled Li anode in the aNi_3_B@CNTs‐based cell exhibited a smooth surface and a much lower sulfur content (17.9 wt.% vs. 23.8 wt.%), which differs significantly to the rough surface with irregular bumps and cracks observed in the cNi_3_B@CNTs‐based cell. Additionally, the separator in the cell with aNi_3_B@CNTs showed minimal color change, whereas the separator with cNi_3_B@CNTs became yellow because of the substantial quantity of LiPSs dissolved in the electrolyte, which indicates worse reaction environments for LiPSs. These analyses demonstrated that the aNi_3_B@CNTs host can effectively anchor soluble LiPSs, accelerates their bidirectional conversion, facilitates homogeneous Li_2_S_2_/Li_2_S deposition, and promotes instant Li_2_S decomposition. This creates a virtuous cycle for achieving high‐performance LSBs. The EIS result after cycling can be used to verify our hypothesis. As shown in Table S1 (Supporting Information), after being fully discharged for 100 cycles, the aNi_3_B@CNTs exhibited a much lower *R*_ct_ value than the cNi_3_B@CNTs, indicating a much smaller internal resistance of the aNi_3_B@CNTs owing to the porous and uniform Li_2_S_2_/Li_2_S deposition. Benefitting from such a virtuous cycle, the aNi_3_B@CNTs‐based cell maintained a high discharge capacity of 254.57 mAh g^−1^ after 2000 cycles, even at an ultrahigh C‐rate of 5 C (Figure 5i), which corresponded to an ultralow capacity decay of 0.028% per cycle, suggesting the impressive cycle life at ultrahigh rates.

**Figure 6:**
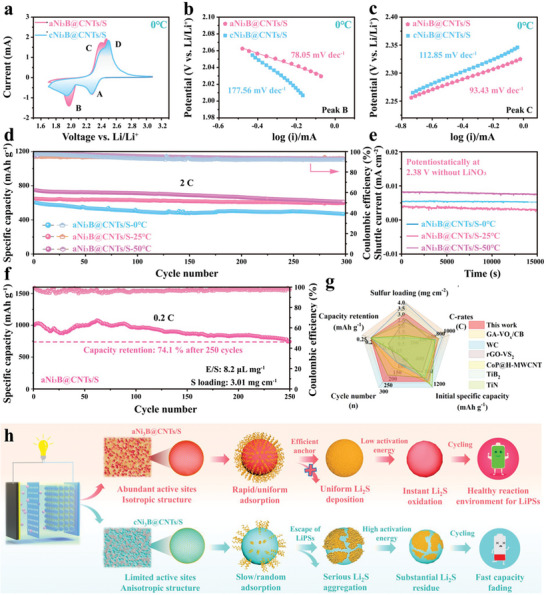
a) CV curves based on 0.1 mV s^−1^ at 0 °C. Tafel plots calculated from b) reduction peak and c) oxidation peak. d) Cyclic performance under 2 C at various temperatures. e) Shuttle current measurements at different temperatures. f) Cyclic performance of aNi_3_B@CNTs‐based cell with a high sulfur loading of 3.01 mg cm^−2^. g) Electrochemical performance comparison with some reported MX and crystalline MB materials. h) Schematic illustration of mechanism by which aNi_3_B@CNTs create favorable reaction environments in LSBs.

Most studies have focused on properties at room temperature, which are far short of the requirements for complicated temperature conditions in actual applications. For LSBs, high temperatures severely aggravate the shuttle behavior of LiPSs owing to their increased solubility in the electrolyte, thus resulting in inferior cyclic performance. At low temperatures, the redox kinetics of LiPSs are considerably hindered; in particular, the solid‐phase reaction of Li_2_S_2_/Li_2_S is more tough due to the slower kinetic velocity and lower reaction voltage, which results in a rapid deterioration in capacity.^[^ ^46^ ^]^ In this study, the electrochemical performances over a wide range of temperatures were investigated and compared. At a low temperature of 0 °C, the aNi_3_B@CNTs‐based cell still demonstrated superior characteristics compared with the cNi_3_B@CNTs‐based cell, including higher current response, distinct anodic peak separation, shifts toward the positive or negative region for reduction/oxidation peaks, and significantly lower Tafel slopes, as illustrated in **Figure** **6**a–c. Similar phenomenon were observed in the high temperature condition of 50 °C (Figure S16a–c, Supporting Information). This suggests that aNi_3_N@CNTs can still enable the rapid conversion of LiPSs at high and low temperatures, thereby minimizing the effect of temperature variation. Accordingly, excellent cycling stability in extreme temperature conditions was achieved. As presented in Figure 6d and Figure S17 (Supporting Information), the aNi_3_B@CNTs‐based cell demonstrated exceptional cyclic capacity decays of only 0.107%, 0.059% and 0.076% at 2 C for 300 cycles, even at −10, 0, and 50 °C, which were comparable to the decay rate at room temperature (0.027%), suggesting promising cyclic stability under extreme working conditions.

Moreover, the aNi_3_B@CNTs‐based cell exhibited close shuttle currents of 0.005, 0.004, and 0.008 mA cm^−2^ at 0, 25, and 50 °C, respectively, verifying the effective suppression of shuttle behavior under different temperature conditions (Figure 6e). Post‐SEM analyses were performed on both electrodes after cycling at extreme temperatures. As shown in Figure S19a–d and i–l (Supporting Information), at both 50 and 0 °C, the aNi_3_B@CNTs electrode still presented a uniform and porous structure after 100 cycles, verifying that the high conversion efficiency of LiPSs were well maintained even at extreme operating environments. In contrast, the cNi_3_B@CNTs/S electrode exhibited significant agglomeration of non‐conductive Li_2_S_2_/Li_2_S and visible cracks after the cyclic process (Figure S19e–h,m–p, Supporting Information). In addition, the post‐TEM and XPS analysis were conducted to investigate the chemical stability of Ni_3_B electrocatalyst. As shown in Figure S20 (Supporting Information) and related discussion, both aNi_3_B and cNi_3_B nanoparticles exhibited satisfactory chemical stability during the cyclic process. However, the anisotropic crystalline Ni_3_B with limited active sites could not withstand the effect of environmental temperature changes on the reaction kinetics of polysulfides; consequently, the polysulfide reaction environment deteriorated gradually during the cyclic process.

High sulfur loading is a crucial requirement for commercialization of LSBs. As displayed in Figure 6f, the aNi_3_B@CNTs/S maintained its outstanding cycling stability at a high sulfur loading of 3.01 mg cm^−2^. A high initial capacity of 1002.85 mAh g^−1^ and capacity retention of 74.10% were achieved after 250 cycles at 0.2 C. At a high sulfur loading of 5.6 mg cm^−2^ and a low E/S ratio of 5.0 ul mg^−1^, as shown in Figure S21 (Supporting Information), the aNi_3_B@CNTs‐based cell also achieved high areal capacities of 5.87, 5.04, 4.64, 3.80 mAh cm^−2^ at 0.1, 0.2, 0.3, and 0.5 C, respectively. Moreover, a high area capacity of 5.60 mAh cm^−2^ was still recovered when reverting the C‐rate back to 0.1 C, demonstrating the superior rate performance. Impressively, when the sulfur loading was further elevated to 10.45 mg cm^−2^ (Figure S22, Supporting Information), the aNi_3_B@CNTs‐based cell achieved a satisfactory initial areal capacity of 9.56 and retained 7.52 mAh cm^−2^ over 40 cycles with a low E/S usage of 3.9 µL mg^−1^, suggesting its promising prospects as sulfur host for practical LSBs. Considering all crucial parameters, including the long‐term cycle life, rate capability, and performance at high sulfur loading, the aNi_3_B@CNTs‐based cell demonstrates multifaceted and superior electrochemical performance compared with previously reported MX or crystalline MB materials (Figure 6g; Figure S23a,b, and Tables S2–S4, Supporting Information). This is endowed by the virtuous cycle for the favorable LiPSs redox environments created by amorphous Ni_3_B. Figure 6h provides a comprehensive illustration of the virtuous cycle process. Exploiting the binary sulfiphilic adsorption associated with a self‐regulation nature, the amorphous Ni_3_B consistently exhibited the highest electrocatalytic facet with high density of anchoring sites for LiPSs, which realizes a rapid and uniform adsorption of LiPSs during the initial stage of the cyclic process. This minimizes electrolyte contamination arising from the accumulation of LiPSs. Meanwhile, the high catalytic capability, abundant active sites, and rapid ion/electron transport synergistically facilitated the rapid/uniform Li_2_S precipitation. The uniform Li_2_S deposition is beneficial for the complete oxidation of Li_2_S to LiPSs. The produced LiPSs are again rapidly and uniformly adsorbed for the next sulfur evolution process, thus creating a virtuous cycle for the future sulfur species conversion.

## 3 Conclusion

We rationally constructed an isotropic host interface to create a virtuous cycle for favorable sulfur species conversion environment. Theoretical simulations and experimental analyses revealed that the aNi_3_B@CNTs could enable a rapid LiPSs conversion and an instant redox of solid Li_2_S, avoiding the accumulation of insulating Li_2_S on the electrode surface. Meanwhile, the binary sulfiphilic adsorption capability of aNi_3_B@CNTs could minimize the electrolyte contamination caused by the escape of LiPSs. Benefitting from these merits, the aNi_3_B@CNTs as a host material created a virtuous cycle for sulfur species conversion during the entire SRR and SER processes. As a result, the aNi_3_B@CNTs‐based cell exhibited high sulfur utilization, remarkable rate performance, and superior cyclic stability. Impressively, it exhibited an ultralong cyclic life even at a high C‐rate of 5 C, demonstrating its high potential for fast‐charging batteries. Under extreme temperature of 0 and 50 °C, the aNi_3_B@CNTs‐based cell maintained the impressive cycling stability, which were even comparable to that tested at room temperature, suggesting its promising prospects for LSBs under harsh temperature working environments. This work proposes the concept of creating a virtuous cycle for the reaction environment of sulfur species to expedite the practical application of LSBs.

## 4 Experimental Section

### Synthesis of aNi_3_B@CNTs

The aNi_3_B@CNTs were synthesized using a one‐step aqueous reduction process. First, 20 mg of commercial carbon nanotubes (CNTs) were added to a NiCl_2_ solution (20 mL, 0.25 M) and followed by a sonication for 30 min. Subsequently, a KBH_4_ solution (20 mL, 1 M) was gradually injected into the vigorously stirred NiCl_2_@CNTs solution (20 mL, 0.25 M) using a microinjection pump for 5 min. The resulting mixture was stirred for 10 min. Next, the aNi_3_B@CNTs was obtained by repeatedly washing with absolute ethanol and then drying overnight. In addition, a cNi_3_B@CNTs composite was obtained by annealing the as‐prepared aNi_3_B@CNTs at 350 °C for 2 h under an Ar protective atmosphere.

### Computational Details

The Vienna Ab initio simulation package (VASP) was used to conduct all spin‐polarized DFT calculations for periodic material systems^[^ ^47^ ^]^ based on the projector‐augmented wave (PAW) method.^[^ ^48^ ^]^ In the spin‐polarized DFT calculations for periodic material systems, the exchange‐correlation function was handled by the generalized gradient approximation (GGA) developed by Perdew–Burke–Ernzerhof (PBE). Additionally, the van der Waals (vdW) interactions were described using Grimme's DFT‐D3 method.^[^ ^49^ ^]^ The projector augmented wave approach was used to describe the interaction between the atomic core and electrons in spin‐polarized DFT calculations for periodic material systems. The energy cutoff for the plane‐wave basis set was chosen to be 500 eV.^[^ ^50^, ^51^ ^]^ For geometry relaxation in all surface structures, the Brillouin zone was sampled with Gamma (Γ) centered Monkhorst‐Pack mesh sampling using a 3 × 3 × 1 mesh. For bulk structures, a 5 × 5 × 5 mesh was used for the Brillouin zone sampling. Vacuum spacing of 15 A sampled was present in all surface slabbed models, ensuring minimal lateral interaction of adsorbates.^[^ ^52^, ^53^ ^]^ The bottom layers about half of the structure were kept frozen at the lattice position. The convergences of energy, gradient in geometry optimization were converged to < 1×10^−6^ eV, 0.02 eV Å^−1^,^[^ ^54^, ^55^ ^]^ respectively. The climbing image nudged‐elastic band (CI‐NEB) method was used to search for transition states.^[^ ^56^ ^]^ The aNi_3_B model, which was constructed using the melt quenching technique, was diffused at 2000 K and then quenched at low temperature using ab initio molecular dynamics (AIMD).^[^ ^57^ ^]^

## Conflict of Interest

The authors declare no conflict of interest.

## Author Contributions

W.X. performed conceptualization, data curation, formal analysis, investigation, methodology, and wrote the original draft. K.Y. performed methodology, resources, supervision, funding acquisition, wrote‐reviewed, and edited the original draft. J.‐H.K. performed conceptualization, supervision, wrote‐reviewed, and edited the original draft. H.X. performed investigation, conceptualization, methodology, validation, reviewed, and edited.

## Supporting information

Supporting Information

## Data Availability

The data that support the findings of this study are available from the corresponding author upon reasonable request.
