# Supplementary material for: Breaking Barriers to High‐Practical Li‐S Batteries with Isotropic Binary Sulfiphilic Electrocatalyst: Creating a Virtuous Cycle for Favorable Polysulfides Redox Environments
Source: Adv Sci (Weinh). 2023 Oct 22;10(33):2303916. doi: 10.1002/advs.202303916 (PMC10667854; doi:10.1002/advs.202303916)
Supplement: Supplementary file 1 — Supporting Information [file ADVS-10-2303916-s001.pdf]

ADVANCED SCIENCE
Open Access

Supporting Information

for *Adv. Sci.*, DOI 10.1002/advs.202303916

Breaking Barriers to High-Practical Li-S Batteries with Isotropic Binary Sulfiphilic Electrocatalyst: Creating a Virtuous Cycle for Favorable Polysulfides Redox Environments

Wei Xiao, Kisoo Yoo, Jong-Hoon Kim* and Hengyue Xu**

Supporting information

Breaking Barriers to High-Practical Li-S Batteries with Isotropic Binary Sulfiphilic Electrocatalyst: Creating a Virtuous Cycle for Favorable Polysulfides Redox Environments

Wei Xiao, Kisoo Yoo, Jong-Hoon Kim*, Hengyue Xu**

W. Xiao, K. Yoo
Department of Mechanical Engineering, Yeungnam University, 280 Daehak-ro, Gyeongsan-si, Gyeongsanbuk-do, 38541, South Korea
E-mail: kisooyoo@yu.ac.kr

J. Kim
Energy Storage and Conversion Laboratory, Department of Electrical Engineering, Chungnam National University, Daejeon, 34134, Republic of Korea
E-mail: qwzxas@hanmail.net

H. Xu

Institute of Biopharmaceutical and Health Engineering, Tsinghua Shenzhen International Graduate School, Tsinghua University, Shenzhen 518055, China

Email: xuhy21@mails.tsinghua.edu.cn

1. Supplementary Experimental Section

Synthesis of aNi_3B@CNTs/S composite

After dissolving 70 mg of sulfur in 20 mL of carbon disulfide, 30 mg of aNi_3B@CNTs

were added to the mixture, which was then homogenized for 30 minutes at room temperature using continuous sonication. In order to create pressure for the incorporation of S into the wrinkles of the aNi_3B@CNTs, the suspension was then kept at 50 °C for 8 hours in a sealed container. Using a rotary evaporator, the carbon disulfide was removed from the homogeneous suspension. The resulting mixture powder was vacuum-dried at 45 °C for 12 hours, producing aNi_3B@CNTs/S composites. To conduct a control experiment, cNi_3B@CNTs/S composites were created using the same procedure.

Material Characterizations

The morphology and microstructure were examined using FESEM (Hitachi SEM-4800, Japan), TEM (Hitachi H-7000, 100KV), and HR-TEM (Tecnai G2 F20 S-TWIN, USA). Elemental mapping and energy-dispersive X-ray spectroscopy (EDS) were performed with the SEM microscope. X-ray diffraction (XRD; PANalytical X-Pert PRO, USA) was utilized to confirm the phase of the material. TGA (SDTQ600, USA) was carried out in N_2 with a 10 °C/min heating rate from room temperature to 600 °C. X-ray photoelectron spectroscopy (XPS) (K-Alpha+, USA) was employed to investigate the bonding characteristics and evaluate the adsorption mechanism. For determining the surface area and pore-size distribution, Brunauer-Emmett-Teller analysis was used.

LSBs assembly and measurements

First, the cathode was prepared by mixing the composite (aNi_3B@CNTs, cNi_3B@CNTs), carbon black, and PVDF powder in a 7:2:1 mass ratio, and dissolving them in NMP to form a homogeneous slurry. The cathode foil was created by coating the slurry onto carbon foil and vacuum-drying it there for an entire night at 60 °C. For LSBs with typical sulfur loading, the mass loading of the active material was controlled to be around 1.0 mg cm^{-2}. With the above electrode serving as the cathode, lithium foil serving as the anode, and Celgard 2300 PP serving as the separator, CR-2032 coin-type cells were assembled. The electrolyte used was 1.0 M LiTFSI in DOL and DME (V/V = 1:1) with 2% $LiNO_3$ additives. Galvanostatic charge/discharge test and cyclic voltammetry measurements were conducted within a voltage window of 1.7-3.1 V. On the Gamry Instrument Warminster (PA, USA), electrochemical impedance spectroscopy (EIS) was carried out in the frequency range of 0.01-10^5 Hz.

Polysulfide absorption test

The Li_2S_6 solution was prepared by dissolving S and Li_2S in a mixed solvent of DME (dimethoxyethane) and DOL (1,3-dioxolane) in a volume ratio of 1:1. The mixture was stirred vigorously at a temperature of 60 ℃ for 24 hours to promote dissolution. For the visual adsorption test, aNi_3B@CNTs and cNi_3B@CNTs powders were added individually to the Li_2S_6 solution and soaked for 2 hours, resulting in aNi_3B@CNTs-Li_2S_6 and cNi_3B@CNTs-Li_2S_6. After 2 hours, the absorption of LiPSs was visually observed. The optical properties of the upper and blank Li_2S_6 solutions were analyzed using UV-vis spectroscopy to investigate changes before and after adsorption. Additionally, the precipitates were collected, dried, and subjected to XPS analysis to determine the chemical composition of the reaction products.

Symmetric-cell assembly and measurements

The electrode was prepared by mixing the host material (aNi_3B@CNTs, cNi_3B@CNTs) and PVDF in a weight ratio of 9:1 in NMP solvent. The resulting slurry was coated onto carbon foil. A 0.5 M Li_2S_6 electrolyte was prepared by mixing S and Li_2S powder with a molar ratio of 5:1 in a mixing solvent of DOL and DME (volume ratio 1:1) with 1 M LiTFSI salt at 60 ℃ for 12 hours in an Ar-filled glove box. Symmetric CR2032 coin cells were assembled with 0.5 M Li_2S_6 electrolyte (25 μL) and two identical above electrodes (mass loading of about 1.0 mg cm-2) as both the working and counter electrode. CV measurement of the symmetric cell was performed over a voltage range of -1.5 to 1.5 V to evaluate polysulfide redox conversion kinetics. EIS was acquired in a frequency range of 0.01 to 10^5 Hz.

Measurements of the Li_2S nucleation/dissolution

A homogenous slurry was prepared by mixing 90 wt% aNi_3B@CNTs and 10 wt% PVDF in NMP solvent under vigorous stirring. The slurry was coated on carbon foil, and then the foil was dried at 60 ℃ for 12 h. Lithium foil and PP were used as the anode and separator, respectively, while the cathode was constructed using aNi_3B@CNTs material. On the cathode side, 20 mL of Li_2S_8 was added, and on the Li anode side, blank electrolyte without Li_2S_8 was added. The cell was discharged at 0.1 mA to 2.06 V galvanostatically to convert Li_2S_8 to Li_2S_6, followed by potentiostatic discharging at 2.05 V to completely convert polysulfides to Li_2S until the current decreased to 1.0×10^{-5} mA. For the Li_2S dissolution test, to fully transform S species into solid Li_2S on catalytically reactive interfaces, the assembled cells

were galvanostatically discharged at 0.112 mA to 1.80 V and then at 0.01 mA to 1.80 V. The cells were then potentiostatically charged at 2.40 V for the oxidation of Li_2S into soluble LiPSs, with the charge ending when the charge current was below 10^{-5} A. The cells were disassembled to observe the morphology of Li_2S deposition/dissolution on both hosts.

Shuttle current measurements

$LiNO_3$-free electrolyte was used for the shuttle current measurement to avoid passivating the lithium anode.[1,2] Prior to galvanostatic charging at 0.2 C to 2.8 V, cells underwent three cycles of discharge-charge. After that, the cells were discharged to 2.38 V and transferred to potentiostatic mode, which is when the shuttle current reached its maximum. When the potentiostatic current reached a steady state, it was referred to as the shuttle current.

2. Supplementary Figures and Tables

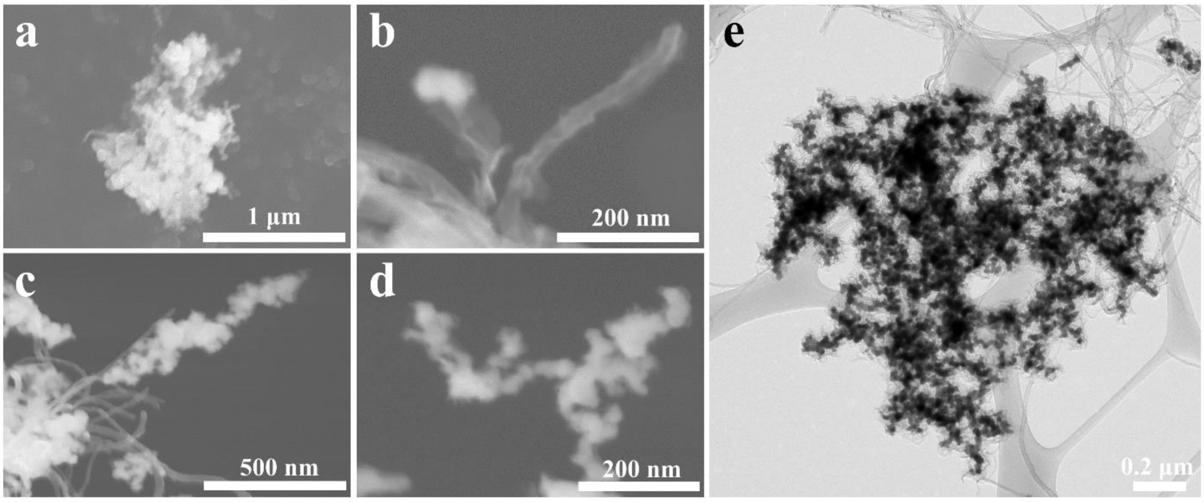

Figure S1. SEM images of a-b) cNi_3B@CNTs, and c-d) cNi_3B@CNTs/S. e) TEM images of cNi_3B@CNTs.

Note: The crystal Ni_3B nanoparticle maintained the structure of amorphous one after calcination, which is used as a control group (**Figure S1**). After sulfur impregnation, the surface of aNi_3B@CNTs/S becomes smooth, indicating the successful loading of sulfur.

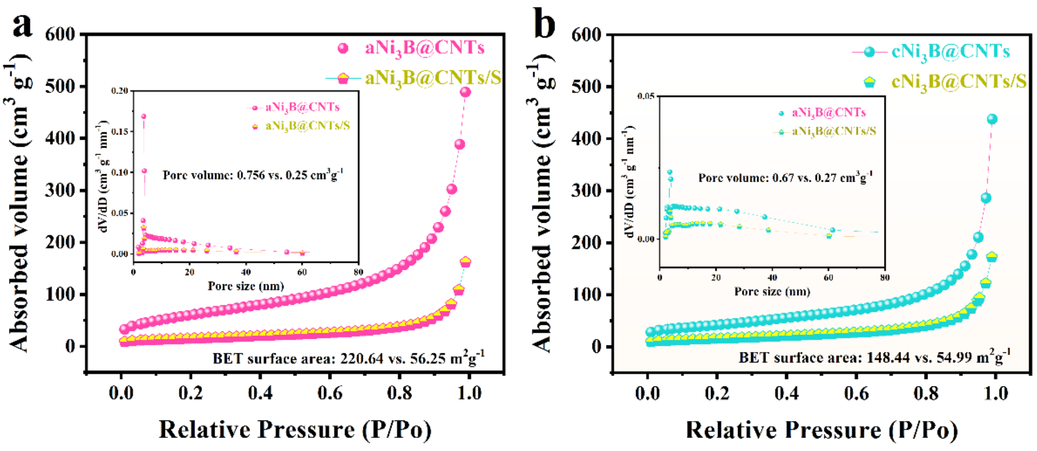

Figure S2. N_2 adsorption/desorption isotherms and pore-size distribution (inset) of a) $aNi_3B@CNTs$, and b) $cNi_3B@CNTs$ before and after sulfur loading.

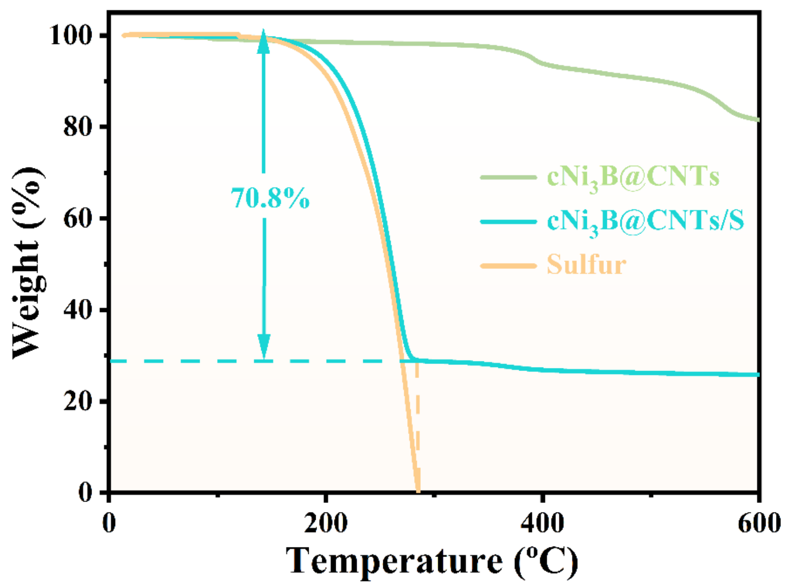

Figure S3. TGA curves of cNi_3B@CNTs and cNi_3B@CNTs/S.

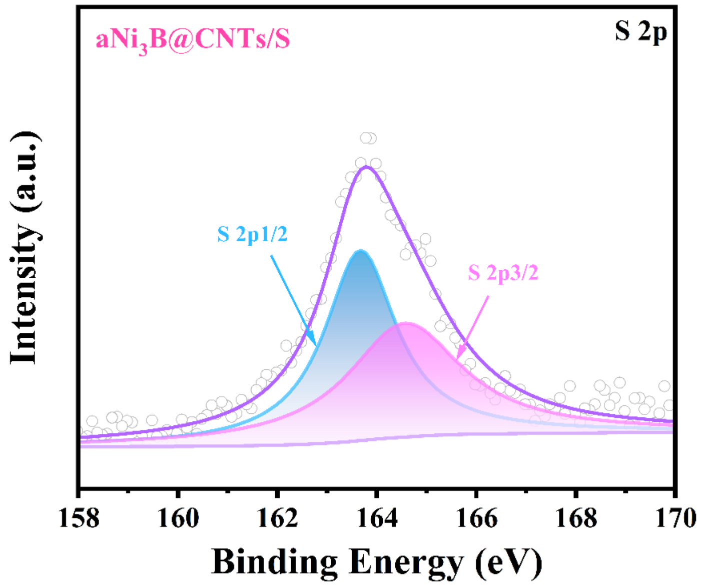

Figure S4. XPS spectra of S2p from aNi_3B@CNTs/S.

Note: The S2p spectra of aNi_3B@CNTs/S are shown in **Figure S4**, and the two peaks at 163.68 and 164.58 eV are attributed to S2p3/2 and S2p1/2, respectively.

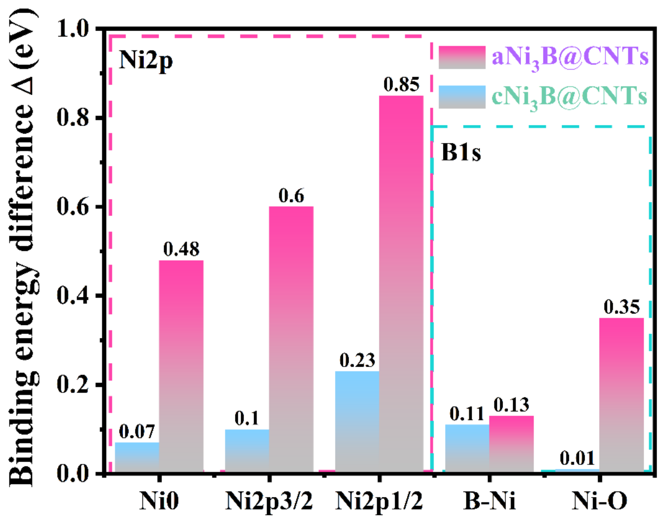

Figure S5. Binding energy shift quantity (Δ) comparison

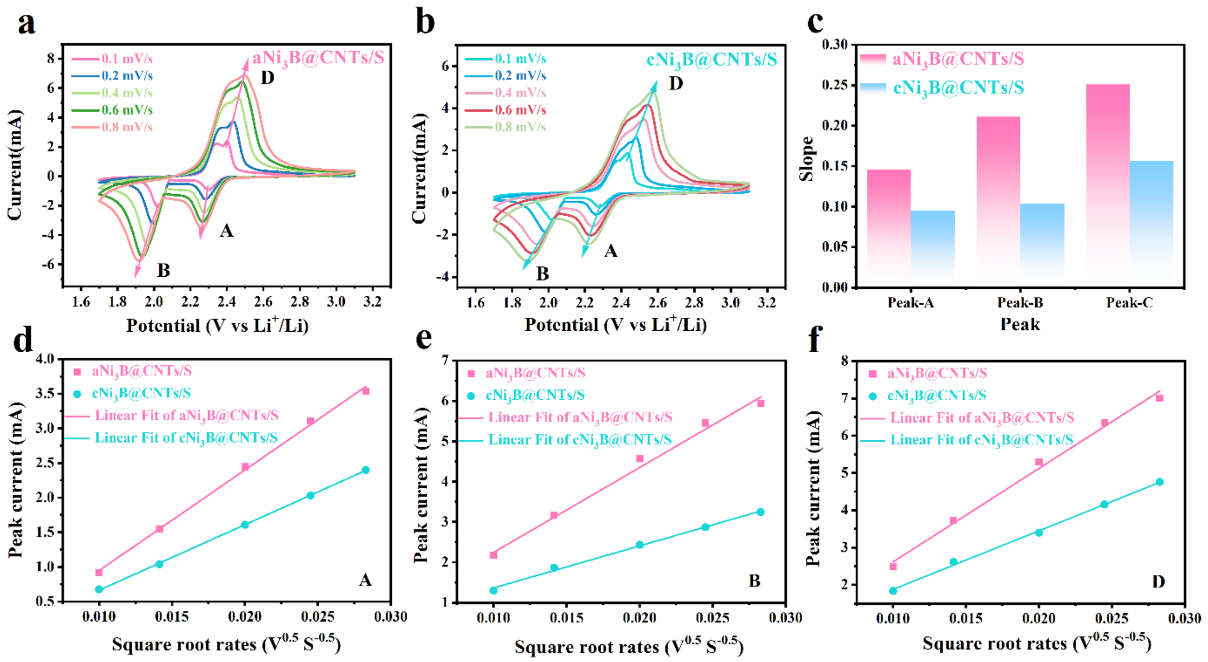

Figure S6. CV curves of LSBs with different host materials: (a) aNi_3B@CNTs, (b) cNi_3B@CNTs in the voltage ranges of 1.7-3.1 V (vs. Li/Li^+) at different scan rates from 0.1 to 0.8 mV s^{-1}. c) Comparison of slope. The corresponding plots of CV peak current of d) Peak A, e) Peak B, and f) Peak C vs. square root of scan rates.

Note: Through CV tests with various scan rates, the Li^+ diffusion kinetics were investigated. (**Figure S6a-b**). The Randles-Sevcik formula was employed to evaluate Li^+ diffusion efficiency, which is given by the equation:[3]

$$\mathrm{Ip} = (2.69 \times 10^5)\, n^{1.5}\, A\, D_{Li^+}^{0.5} C_{Li^+} v^{0.5}$$

where Ip is the peak current, n is the electron charge number, A is the electrode area, D_{Li+} is the diffusion coefficient of Li^+, C is the Li^+ concentration in the cathode, and v is the scan rate. Since n, A, C, and v are known, Ip and $v^{0.5}$ are correlated linearly, and the slope of the curve (Ip-$v^{0.5}$) is positively correlated with D_{Li}^{+}. The slope of aNi_3B@CNTs/S was steeper compared to that for cNi_3B@CNTs/S (as shown in Figure S6c). This indicates that aNi_3B@CNTs exhibited a faster Li^+ diffusion rate, which facilitated the conversion of polysulfides throughout the entire charge/discharge process.

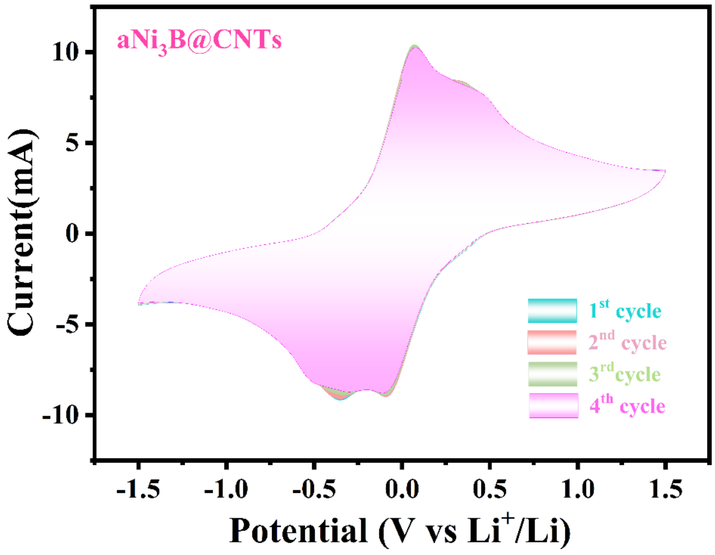

Figure S7. CV curves of symmetric cells with the aNi_3B@CNTs electrode after the four three cycles.

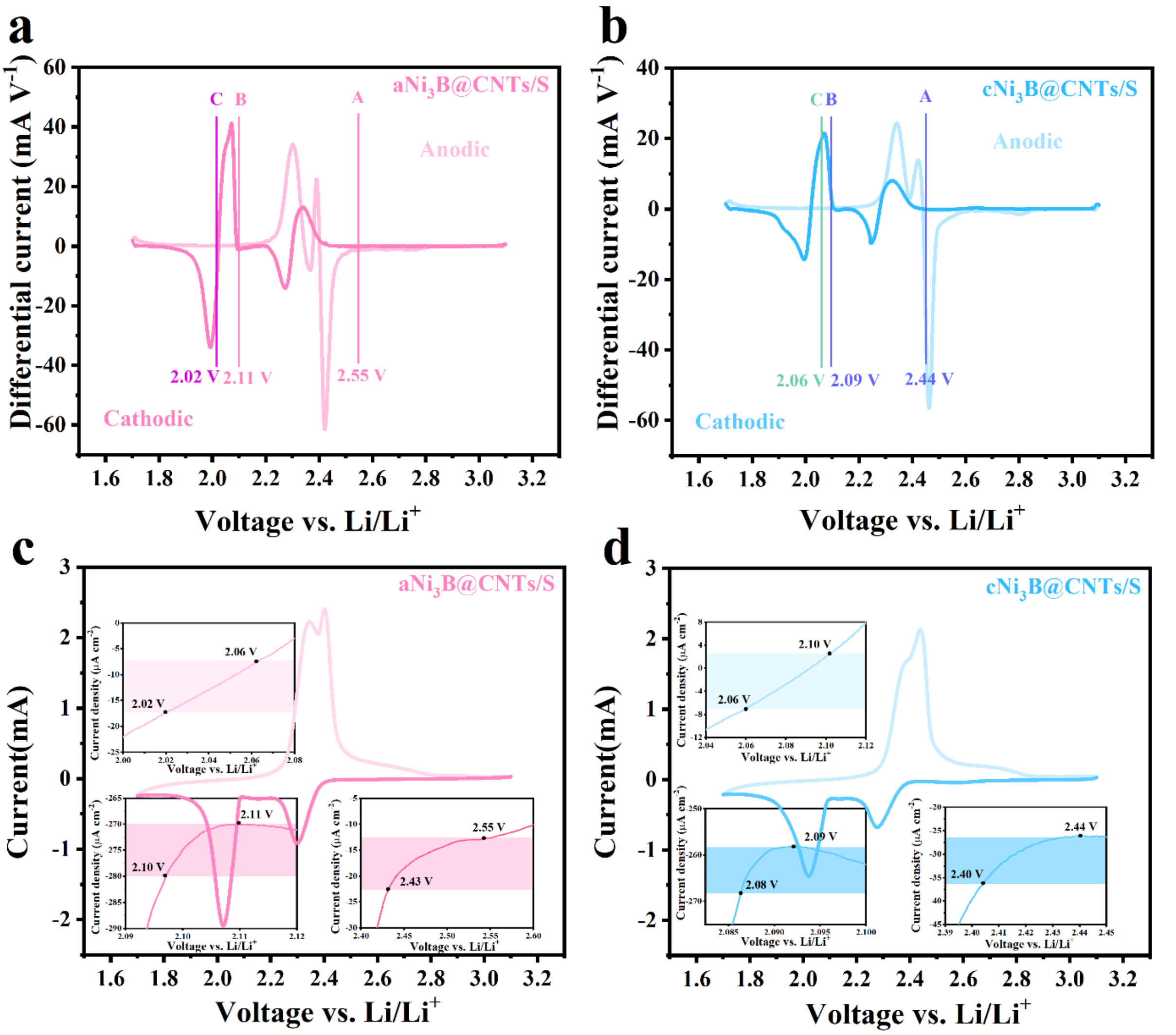

Figure S8. Onset potential tests of the redox process for Li-S cells with a, c) aNi_3B@CNTs, and b, d) cNi_3B@CNTs.

Note: When the change in current density is the smallest, or when dI/dV=0, the baseline voltage and current density are determined as the values preceding the redox peaks. The onset current density and corresponding CV curves are 10 μA cm^{-2} beyond the corresponding baseline current density. [4,5] Specifically, for the cathodic peaks, the onset current density is 10 μA cm^{-2} more negative than the baseline current density, while for the anodic peak, it is 10 μA cm^{-2} more positive than the baseline current density. The inset shows the baseline voltages, with the colored region indicating the current density gap of 10 μA cm^{-2}.

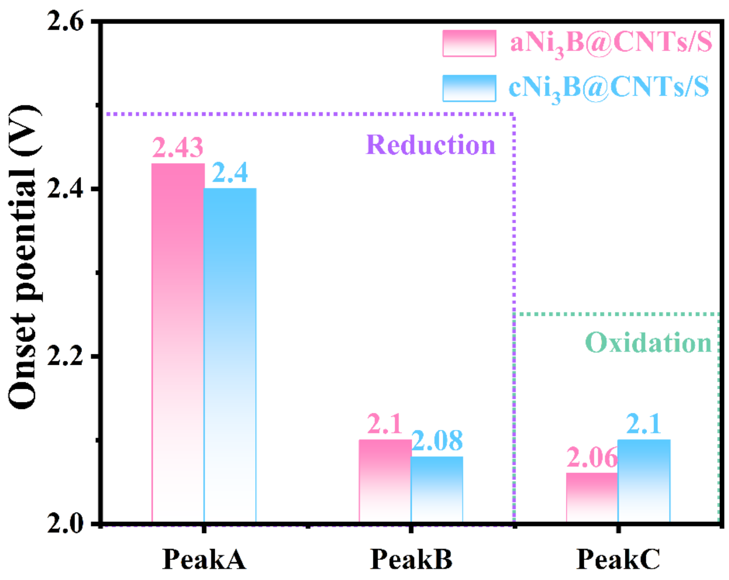

Figure S9. Onset potentials comparison between aNi_3B@CNTs-based and cNi_3B@CNTs-based cell.

Note: Onset potential is defined as the point where the current density surpasses the baseline current density by 10 μA cm^{-2}. This definition is used to evaluate the electrocatalytic activity of aNi_3B@CNTs by observing the variations in cathodic/anodic peaks. As shown in **Figure S9**, the aNi_3B@CNTs-based cell demonstrates the earliest onset potential for redox compared to cNi_3B@CNTs-based cell. This indicates that the amorphous Ni_3B electrocatalyst enhances the kinetics of the redox reaction and significantly reduces polarization of the Li-S cell.

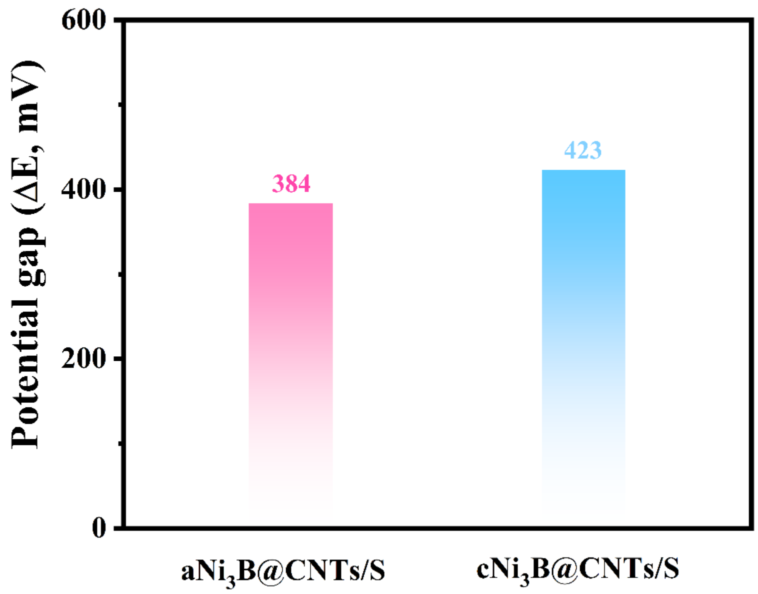

Figure S10. Potential gap (ΔE) comparison.

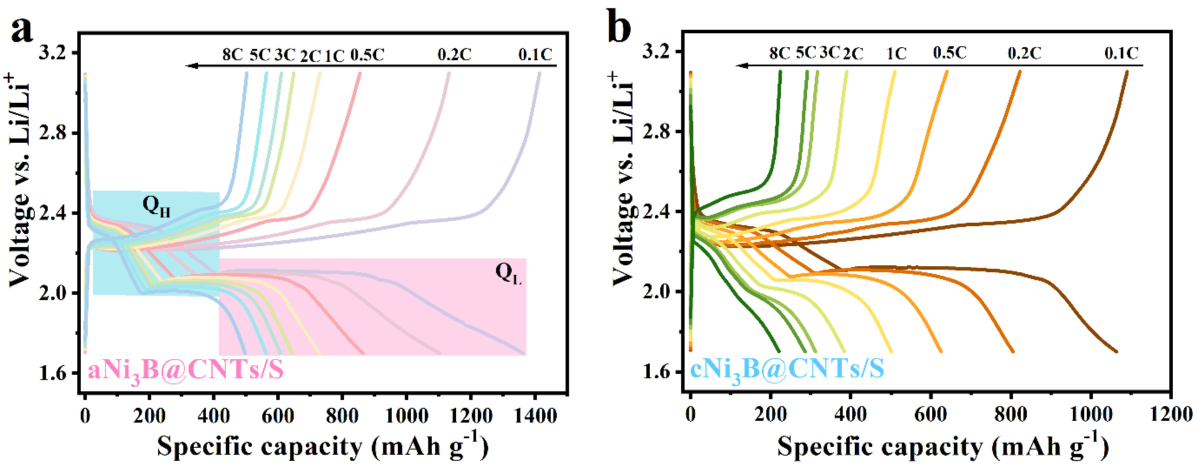

Figure S11. Charge-discharge curves at various current densities of a) aNi_3B@CNTs-based cell, and b) cNi_3B@CNTs-based cell.

Note: As shown in **Figure S11a**, the reduction of sulfur to soluble long-chain LiPSs was associated to the discharge capacity (Q_H) in the high-voltage region, whereas the capacity (Q_L) in the low-voltage plateaus corresponded to the ongoing reduction of LiPSs to insoluble Li_2S_2/Li_2S. The aNi_3B@CNTs-based cell exhibited two distinct discharge plateaus even at an ultra-high C-rate of 8C, indicating that the efficient adsorption of LiPSs and their fast-redox kinetics were successfully achieved with the help of amorphous Ni_3B. Conversely, the second discharge plateaus were notably absent under high C-rates in the cNi_3B@CNTs-based cell (Figure S11b), suggesting severe shuttle behavior of LiPSs and active material loss.

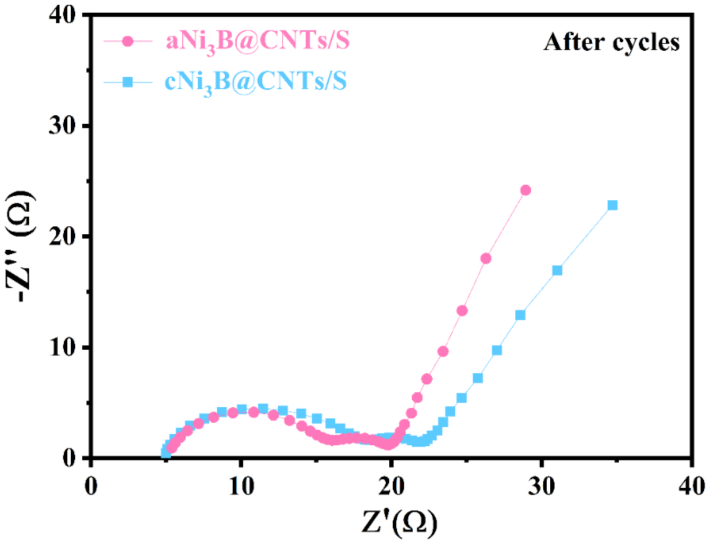

Figure S12. EIS curves comparison after cycling.

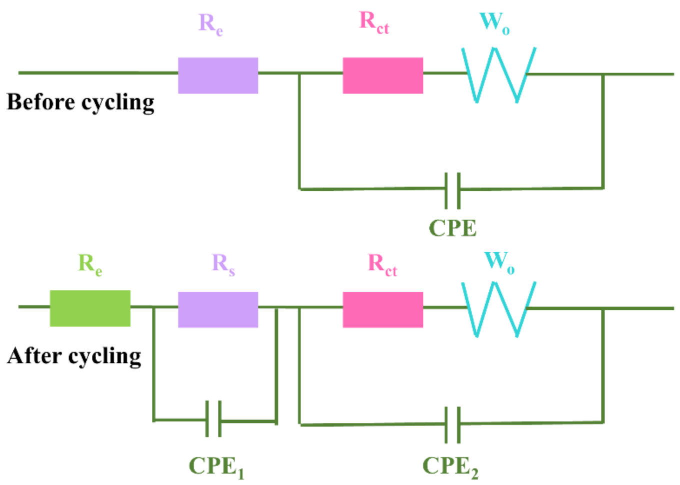

Figure S13. The fitted Randles EIS equivalent circuits of Li-S cells a) before cycling, and b) after cycles.[3]

Note:

R_e: The internal resistance of the electrolyte;

R_s: The resistance of solid electrolyte interfaced film related to solid Li_2S_2/Li_2S;

R_{ct}: The charge-transfer resistance;

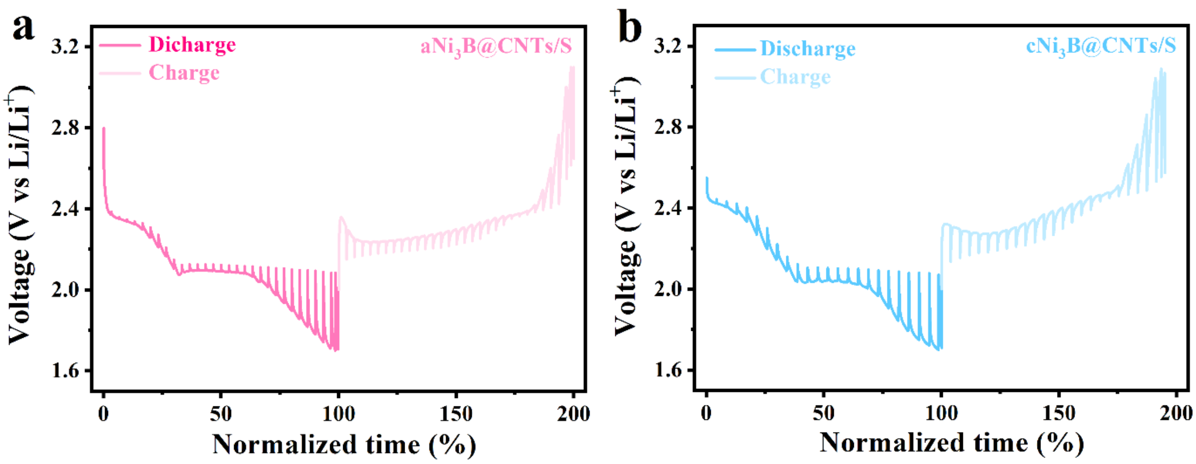

Figure S14. GITT profiles of a) aNi_3B@CNTs-based cell, and b) cNi_3B@CNTs-based cell.

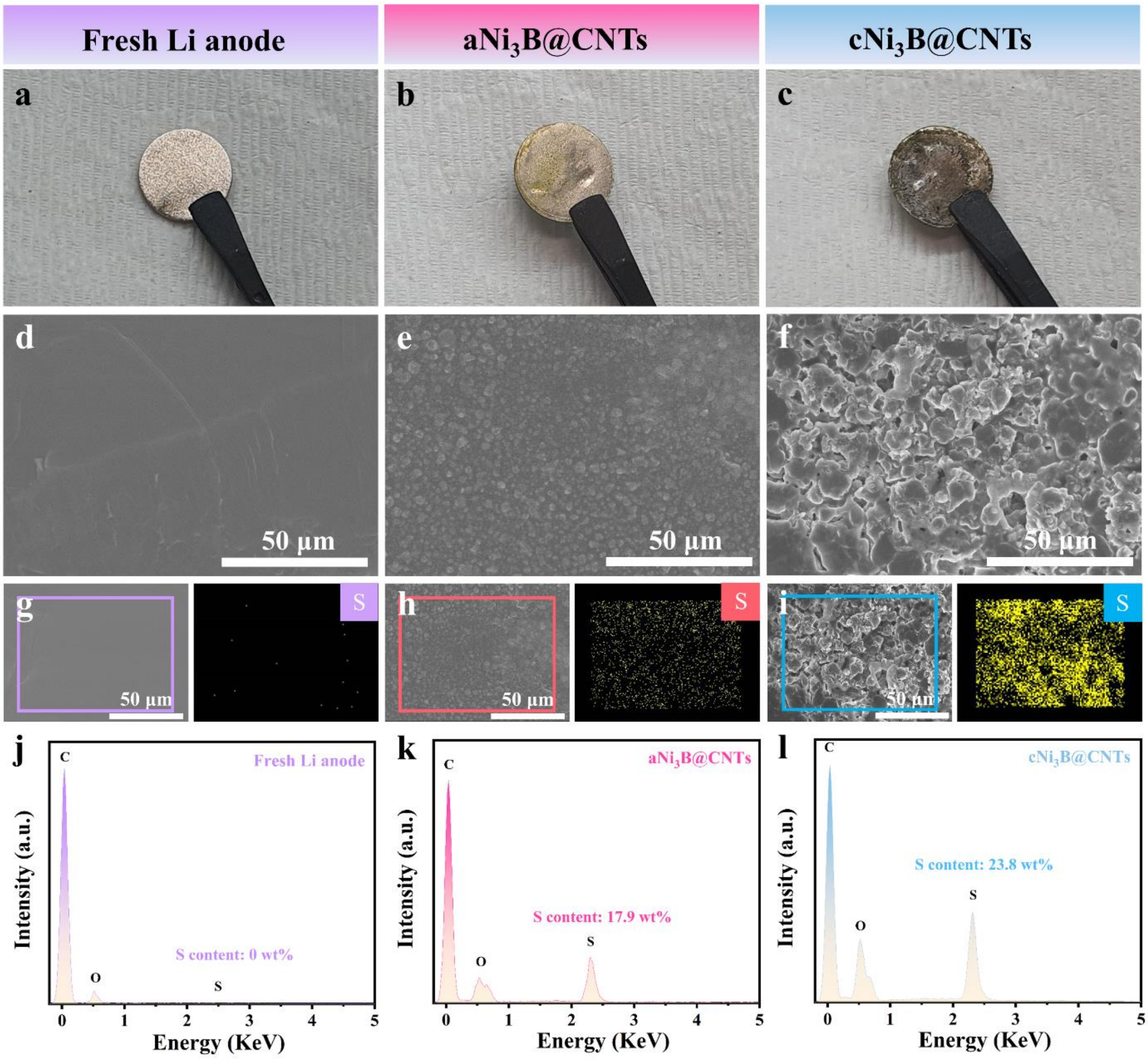

Figure S15. Postmortem studies of the cycled lithium anodes. a-c) Digital pictures. d-f) SEM images. g-i) elemental mapping of sulfur. j-l) EDS analysis.

Note: As shown in **Figure S15b, e**, the Li anode in aNi_3B@CNTs-based cell exhibited a smooth surface with only slight fluffiness, which is even similar to that of fresh lithium metal (Figure S15 a, d). This further signifies that amorphous Ni_3B could effectively suppress polysulfide shuttling, therefore protecting Li anode. In sharp contrast, the lithium anode in cNi_3B@CNTs-based cell showed serious corrosion on the surface due to the parasitic reaction between the migrated polysulfides and anodic lithium. Meanwhile, Li anode in aNi_3B@CNTs-based cell displayed a much lower sulfur content compared to cNi_3B@CNTs-based cell (17.9 vs. 23.8 wt%, Figure S15k-l). These results suggest that aNi_3B@CNTs successfully suppressed the shuttle behavior of LiPSs during the cyclic process, resulting in a favorable reaction environment for LiPSs.

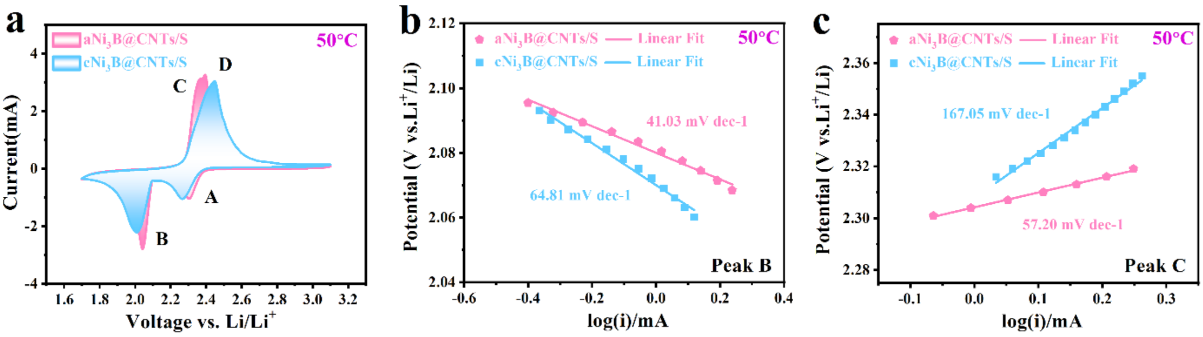

Figure S16. a) CV profiles comparison tested at 50 ℃ with a scan rate of 0.1 mV s^{-1}, and corresponding Tafel plots of b) peak B, and c) and peak C.

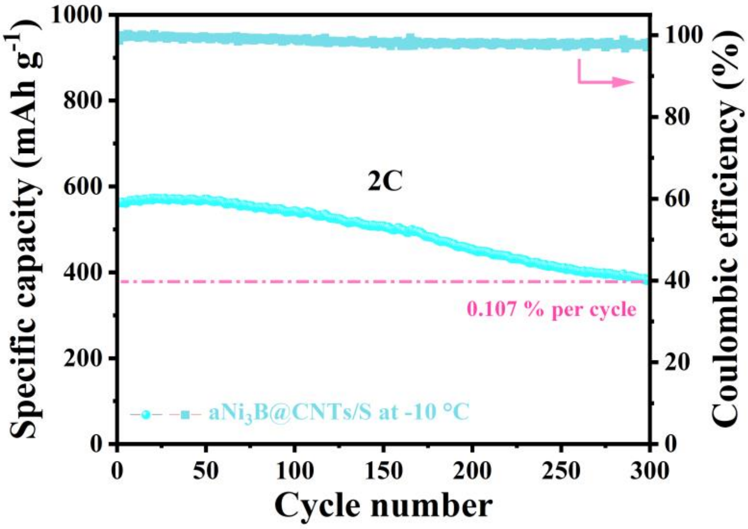

Figure S17. The cyclic performance of aNi_3B@CNTs-based cell at 2C under 10 ℃.

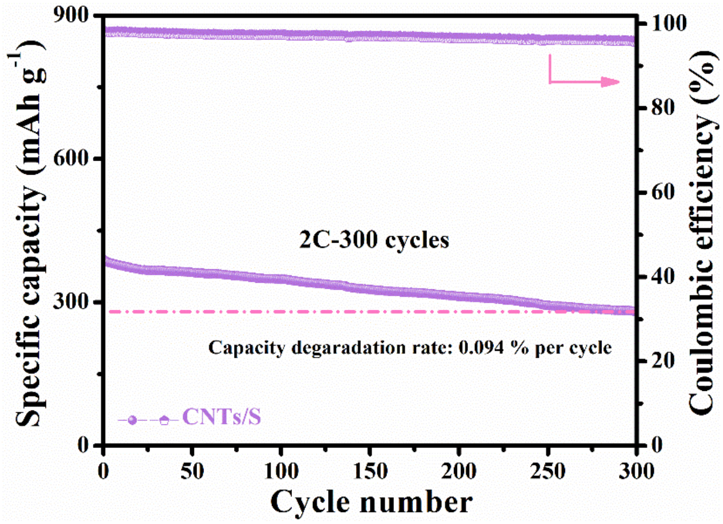

Figure S18. The cyclic performance of CNTs-based cell at 2C for 300 cycles.

As illustrated in **Figure S18**, the CNTs-based cell exhibited a low specific capacity of 389.69 mAh g^{-1}, with only 279.89 mAh g^{-1} of capacity remaining after 300 cycles, corresponding to a rapid capacity degradation rate of 0.094% per cycle. This is in sharp contrast to the high specific capacity (745.20 mAh g^{-1}) and low degradation rate (0.027% per cycle) of aNi$_3$B@CNTs-based cell at 2 C. Such a significant contrast arises from the weak adsorption ability and catalytic capability of CNTs towards the LiPSs conversion process. The prevalent shuttle behavior of LiPSs further contributes to the low sulfur utilization and poor cyclic stability in the CNTs-based cell.

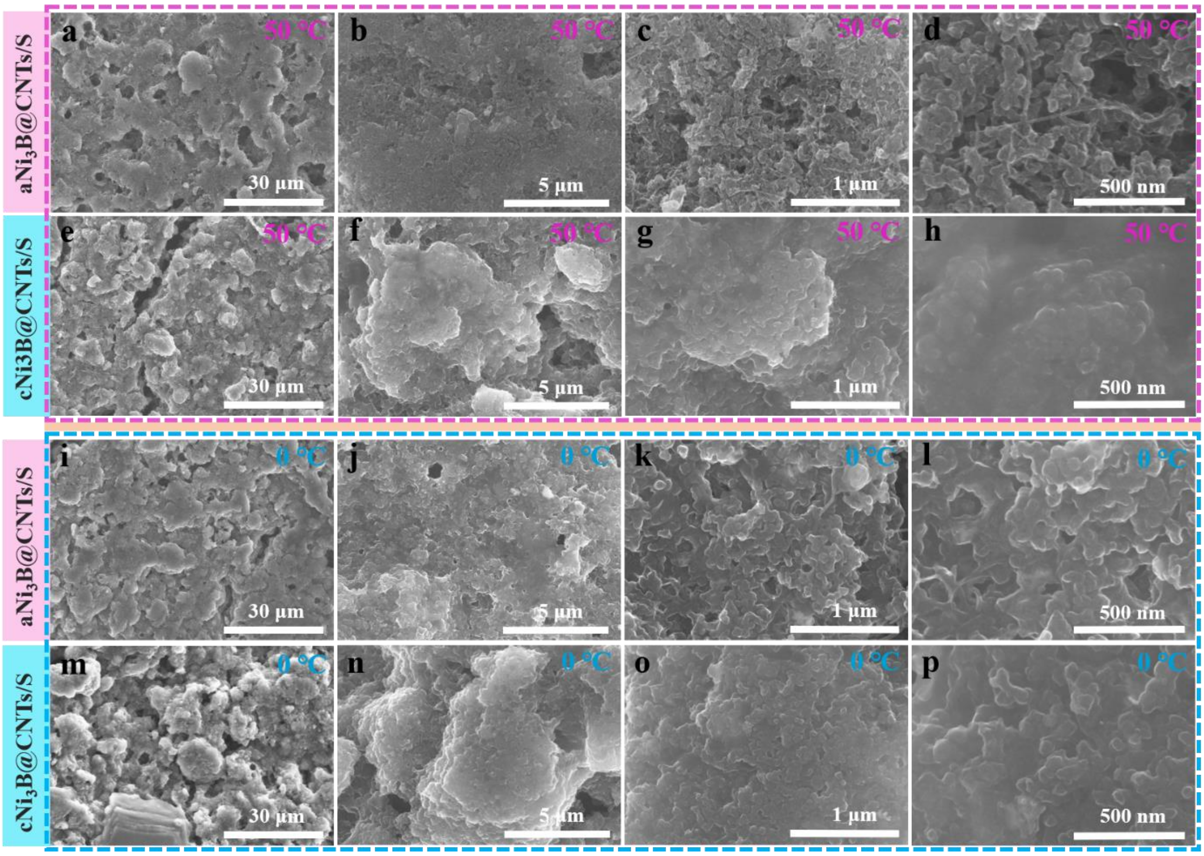

Figure S19. Post-SEM images of a-d) aNi_3B@CNTs/S electrode, and e-h) cNi_3B@CNTs/S after cycling under 50 ℃. i-l) aNi_3B@CNTs/S electrode, and m-p) cNi_3B@CNTs/S after cycling under 0 ℃.

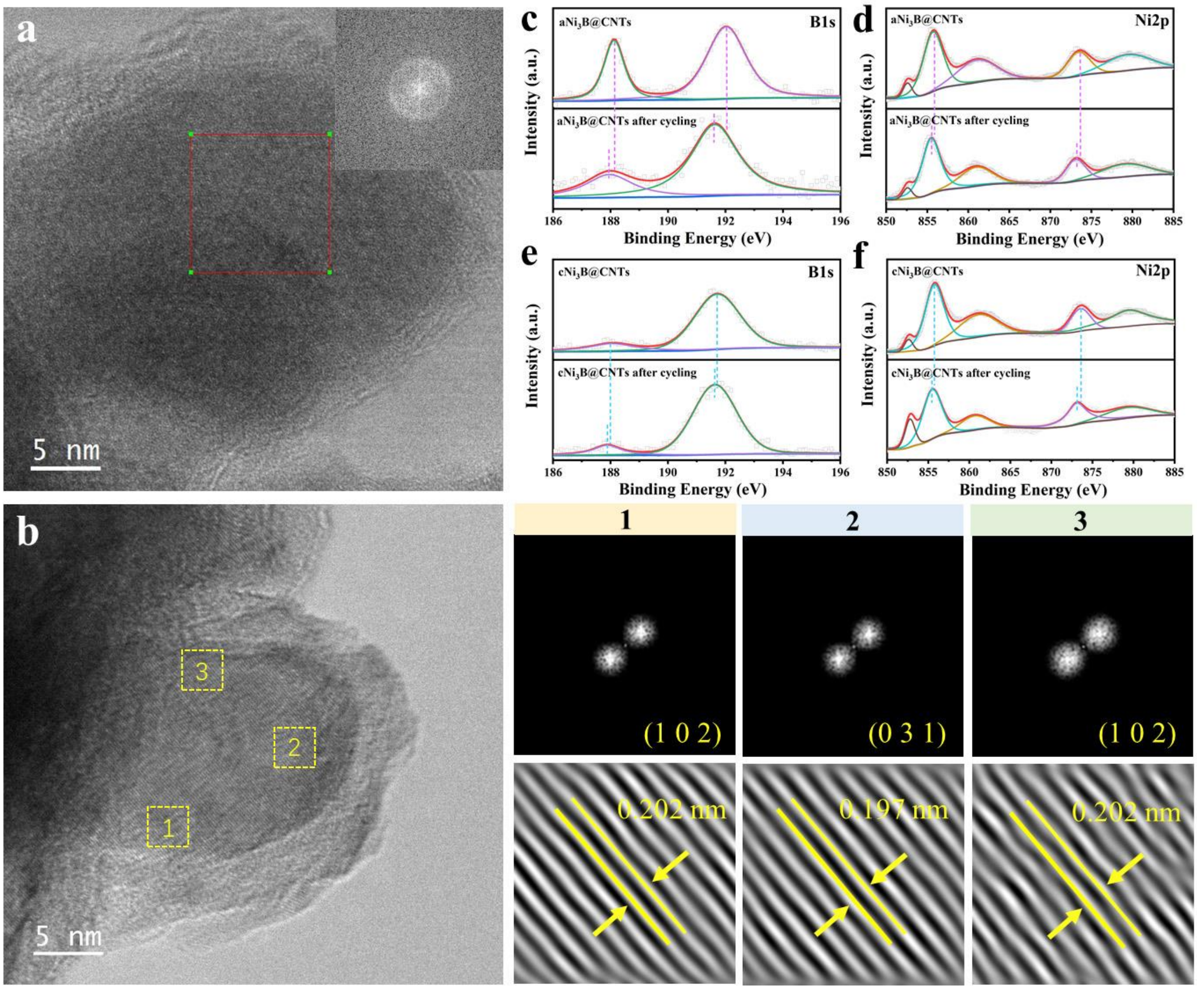

Figure S20. Post HR-TEM image of a) aNi_3B@CNTs after cyclic process, and b) cNi_3B@CNTs after cyclic process. Several Fast Fourier transform (FFT)/inverse FFT pictures of the selected square regions are shown on the right; Post-XPS spectra of c) B1s, and d) Ni2p from aNi_3B@CNTs; e) B1s, and f) Ni2p from cNi_3B@CNTs electrocatalyst comparison before and after cyclic process.

High-resolution transmission electron microscopy (HRTEM) analysis were conducted on the Ni_3B nanoparticles after electrochemical cycling. As shown in **Figure S20a**, the aNi_3B nanoparticles well maintained their amorphous structure, and no phase transference was observed after cyclic process. A similar result was observed in the HRTEM image of cNi_3B nanoparticles. As represented by Figure S20b and related FFT/inverse FFT pictures of the selected region #1-3, there is a main lattice fringe with a d-spacing of ~0.202 nm across the whole cNi_3B particle, which can be ascribed to cNi_3B (102) fringe. And all lattice fringes from selected regions were ascribed to the cNi_3B nanoparticles, suggesting the satisfactory

chemical stability of cNi_3B during the cyclic process. Moreover, the post-XPS analysis further verified this result. As shown in Figure S20 c-f, both Ni2p and B1s spectrum from aNi_3B or cNi_3B slightly shift to a lower binding energy after cycling, which might be ascribed to the interaction between aNi_3B and LiPSs during the cyclic process. More importantly, all those peaks in Ni2P and B1s can still be assigned to Ni_3B, and no additional peak was observed, which further provides intuitive evidence to verify the good chemical stability of the Ni_3B electrocatalyst during the cyclic process. Benefiting from the unique binary sulfiphilic interactions and high catalytic activity, the Ni_3B nanoparticles exhibited highly efficient electrocatalytic effect towards the bidirectional conversion of LiPSs. Due to the instant conversion of LiPSs, the absence of polysulfides-rich environment may lead to minimal side reactions or other chemical transformations.

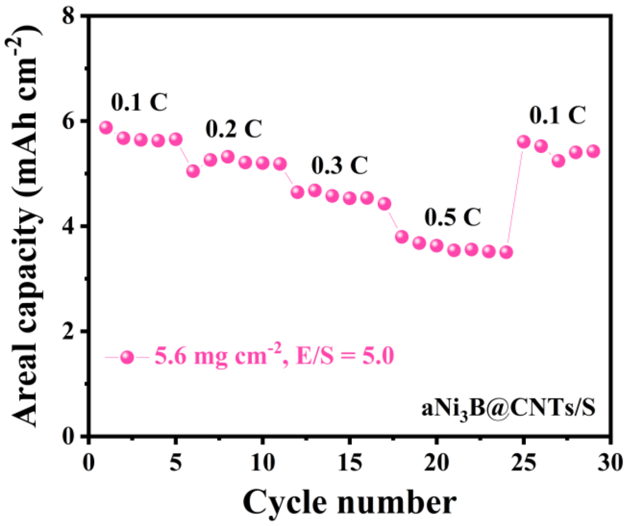

Figure S21. Rate performance of aNi_3B@CNTs-based cell at high sulfur loading.

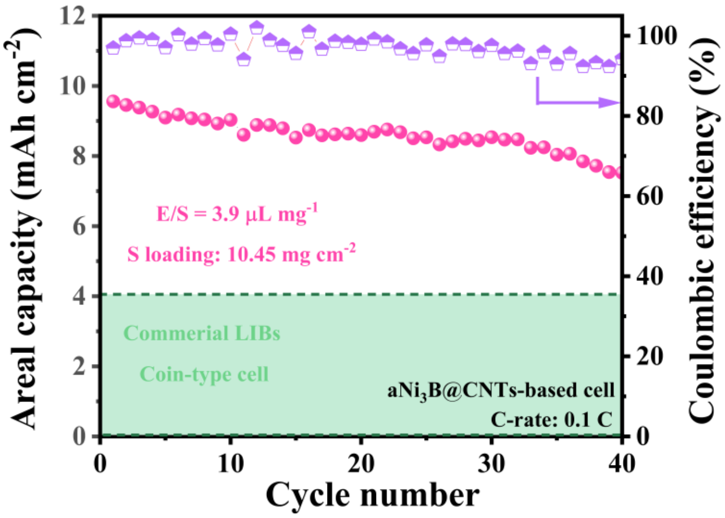

Figure S22. Cyclic performance of aNi_3B@CNTs-based cell at ultra-high sulfur loading.

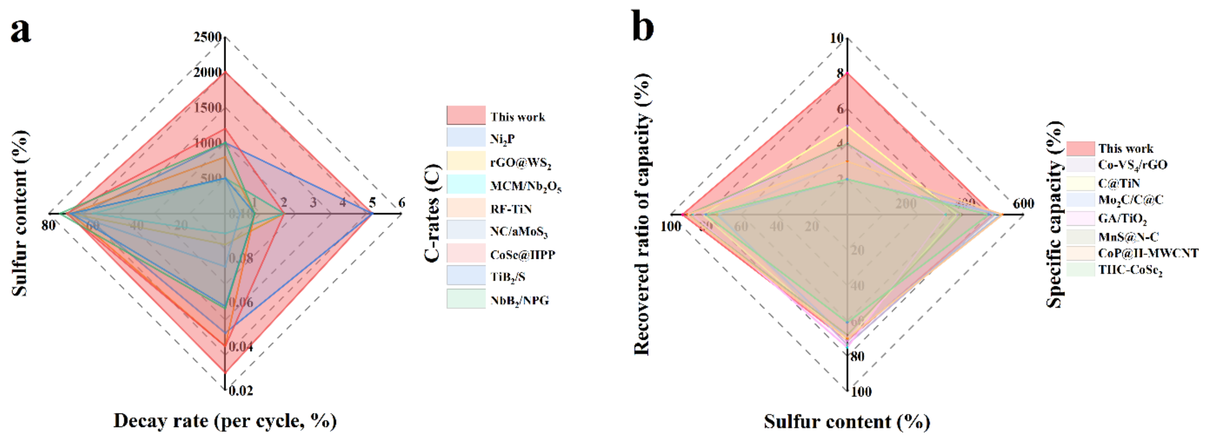

Figure S23. Comparison of a). Cycle life, and b) Rate capability with previous reported materials.

Table S1. The fitted EIS results of Li-S batteries before and after cycling.

Samples	Before cycling		After cycling		
	R_e/Ω	R_{ct}/Ω	R_e/Ω	R_{ct}/Ω	R_s/Ω
aNi_3B@CNTs/S	7.78	26.58	5.08	10.23	4.07
cNi_3B@CNTs	6.09	66.65	4.76	12.15	4.97

Table S2. Corresponding comparison of cyclic performance at high sulfur loading with previous reported materials.

Host material	Sulfur loading (mg cm^{-2})	C-rates (C)	Initial specific capacity	Cycle number	Capacity retention	Ref.
**aNi_3B@CNTs**	**3.03**	**0.2**	**1036.0**	**250**	**778.0**	**This work**
GA-VO_x/CB	2.60	0.2	1057.0	140	630.0	[6]
WC	1.00	0.2	843.0	100	691.3	[7]
rGO–VS_2	2.56	0.1	721.5	100	596.0	[8]
CoP@HPCN-MWCNT	2.30	0.2	887.0	200	753.1	[9]
TiB_2	3.90	0.2	965.0	100	837.0	[10]
TiN	1.20	0.2	1159.4	50	660.0	[11]

Table S3. Corresponding comparison of cyclic life with previous reported materials.

Host material	Sulfur content (%)	C-rates (C)	Cycle number	Capacity decay rate (%)	Ref.
**aNi_3B@CNTs**	**71.2**	**5**	**2000**	**0.033**	**This work**
Ni_2P	65.1	5	1000	0.046	[12]
rGO/WS_2	70.0	2	500	0.086	[13]
MCM/Nb_2O_5	60.0	2	500	0.091	[14]
RF-TiN	70.0	1	800	0.040	[15]
TiB_2/S	70.0	1	500	0.058	[10]
NC/aMoS_3	70.0	0.5	500	0.076	[16]
NbB_2/NPG	75.0	1	1000	0.057	[17]
CoSe@HPP	72.0	2	1200	0.040	[18]

Table S4. Corresponding comparison of rate capability with previous reported materials.

Host material	Sulfur content (%)	C-rates (C)	Specific capacity (mAh g^{-1})	Recovered ratio of capacity (%)	Ref.
**aNi_3B@CNTs**	**71.2**	**5**	**562.37**	**93.33**	**This work**
		**8**	**497.14**		
3%Co-VS_4/rGO	73.6	3	500.00	72.83	[19]
C@TiN	71.0	5	373.00	81.81	[20]
Mo_2C/C@C (2)	70.1	2	520.00	73.76	[21]
GA/TiO_2	75.1	4	333.00	87.42	[22]
MnS@N-C	68.0	4	390.10	90.38	[23]
CoP@HPCN-MWCNT	70.0	3	527.70	90.72	[9]
THC-$CoSe_2$	61.0	2	480.00	80.11	[24]

References

[1] Y. Yao, H. Wang, H. Yang, S. Zeng, R. Xu, F. Liu, P. Shi, Y. Feng, K. Wang, W. Yang, X. Wu, W. Luo, Y. Yu, *Adv. Mater.* **2020**, *32*, 1905658.

[2] C. Y. Chen, H. J. Peng, T. Z. Hou, P. Y. Zhai, B. Q. Li, C. Tang, W. Zhu, J. Q. Huang, Q. Zhang, *Adv. Mater.* **2017**, *29*, 1606802.

[3] S. Hu, X. Huang, L. Zhang, G. Li, S. Chen, J. Zhang, X. Liu, *Adv. Funct. Mater.* **2023**, 2214161.

[4] Z. Yuan, H. J. Peng, T. Z. Hou, J. Q. Huang, C. M. Chen, D. W. Wang, X. B. Cheng, F. Wei, Q. Zhang, *Nano Lett.* **2016**, *16*, 519.

[5] W. Yao, W. Zheng, J. Xu, C. Tian, K. Han, W. Sun, S. Xiao, *ACS Nano* **2021**, *15*, 7114.

[6] Y. Zhang, X. Ge, Q. Kang, Z. Kong, Y. Wang, L. Zhan, *Chem. Eng. J.* **2020**, *393*, 124570.

[7] J. Choi, T. G. Jeong, B. W. Cho, Y. Jung, S. H. Oh, Y. T. Kim, *J. Phys. Chem. C* **2018**, *122*, 7664.

[8] Z. Cheng, Z. Xiao, H. Pan, S. Wang, R. Wang, *Adv. Energy Mater.* **2018**, *8*, 1702337.

[9] Z. Ye, Y. Jiang, J. Qian, W. Li, T. Feng, L. Li, F. Wu, R. Chen, *Nano Energy* **2019**, *64*, 103965.

[10] C. Li, X. Liu, L. Zhu, R. Huang, M. Zhao, L. Xu, Y. Qian, *Chem. Mater.* **2018**, *30*, 6969.

[11] T. G. Jeong, D. S. Choi, H. Song, J. Choi, S. A. Park, S. H. Oh, H. Kim, Y. Jung, Y. T. Kim, *ACS Energy Lett.* **2017**, *2*, 327.

[12] J. Cheng, D. Zhao, L. Fan, X. Wu, M. Wang, N. Zhang, K. Sun, *J. Mater. Chem. A Mater.* **2017**, *5*, 14519.

[13] X. Li, Z. Pan, Z. Li, X. Wang, B. Saravanakumar, Y. Zhong, L. Xing, M. Xu, C. Guo, W. Li, *J. Power Sources* **2019**, *420*, 22.

[14] Y. Tao, Y. Wei, Y. Liu, J. Wang, W. Qiao, L. Ling, D. Long, *Energy Environ. Sci.* **2016**, *9*, 3230.

[15] Z. Xing, G. Li, S. Sy, Z. Chen, *Nano Energy* **2018**, *54*, 1.

[16] J. Yu, J. Xiao, A. Li, Z. Yang, L. Zeng, Q. Zhang, Y. Zhu, L. Guo, *Angew. Chem. Int. Ed.*

2020, *59*, 13071.

[17] B. Wang, L. Wang, B. Zhang, S. Zeng, F. Tian, J. Dou, Y. Qian, L. Xu, *ACS Nano* **2022**, *16*, 4947.

[18] Z. Ye, Y. Jiang, L. Li, F. Wu, R. Chen, *Adv. Mater.* **2020**, *32*, 2002168.

[19] Y. Dong, R. Zhang, H. Peng, D. Han, X. Zheng, Y. Han, J. Zhang, *ACS Appl. Mater. Interfaces* **2022**, *14*, 32474.

[20] Y. Wang, R. Zhang, Y. chao Pang, X. Chen, J. Lang, J. Xu, C. Xiao, H. Li, K. Xi, S. Ding, *Energy Storage Mater.* **2019**, *16*, 228.

[21] Z. Wang, X. Xu, Z. Liu, S. Ji, S. O. Ahmed Idris, J. Liu, *Electrochim. Acta* **2020**, *332*, 135482.

[22] J. Q. Huang, Z. Wang, Z. L. Xu, W. G. Chong, X. Qin, X. Wang, J. K. Kim, *ACS Appl. Mater. Interfaces* **2016**, *8*, 28663.

[23] F. Ma, P. Hu, T. Wang, J. Liang, R. Han, J. Han, Q. Li, *ACS Appl. Energy Mater.* **2021**, *4*, 3487.

[24] B. Guo, Q. Ma, L. Zhang, T. Yang, D. Liu, X. Zhang, Y. Qi, S. J. Bao, M. Xu, *Chem. Eng. J.* **2021**, *413*, 127521.
